# Visible trephine-based foraminoplasty in PTED leads to asymmetrical stress changes and instability in the surgical and adjacent segments: a finite element analysis

**DOI:** 10.1186/s13018-023-03916-0

**Published:** 2023-06-13

**Authors:** Wenliang Wu, Ruixuan Yu, Hongkai Hao, Kaiyun Yang, Guangjun Jiao, Haichun Liu

**Affiliations:** 1 Department of Spine Surgery, Qilu Hospital of Shandong University, Wenhuaxi Road No.107, Jinan, 250012 People’s Republic of China; 2 Institute of Stomatology, Qilu Hospital of Shandong University, Jinan, 250012 People’s Republic of China; 3 Department of Physician Training, Qilu Hospital of Shandong University, Jinan, People’s Republic of China

**Keywords:** Percutaneous endoscopic transforaminal discectomy, Foraminoplasty, Visible trephine, Finite element analysis

## Abstract

This study aimed to construct a multi-segment lumbar finite element model (FEM) of PTED surgery to analyze the changes in stress and ROM after visible trephine-based foraminoplasty. The CT scans of a 35-year-old healthy male were used to develop a multi-segment lumbar FEM with Mimic, Geomagic Studio, Hypermesh and MSC.Patran. Different foraminoplasty was performed on the model, and these were grouped into normal group (A), the ventral resection group (B), the apex resection group (C), the ventral + apex + isthmus resection group (D), and the SAP + isthmus + lateral recess resection group (E). A vertical load of 500N and a torque of 10N·M were applied to the upper surface of the L3 vertebral body to simulate the biomechanical characteristics under the motion of flexion, extension, lateral bending, and rotation. The von Mises stress maps of the intervertebral f, vertebral body, facet joints, and the ROM of the L3-S1 intervertebral disk were calculated and analyzed. The changes of peak stress on the vertebral body for each group were not significant in the same motion state. Significant stress differences were observed in the L4/5 intervertebral disks, while no obvious stress changes were observed for the L3/4 and L5/S1 intervertebral disks. The stress of the L3/4 and L5/S1 facet joints decreased after L4/5 foraminoplasty, while the stress of L4/5 facet joints displayed an overall increasing trend. Significant asymmetrical stress changes of bilateral facet joints were observed in all three segments, particularly during bilateral rotation movements. The ROM of L3-S1 gradually increased from Group A to Group E, especially during flexion, left lateral bending, and right rotation, with the highest elevation observed for the L45 ROM. Our FEM indicated that enlarged resection and exposure of the articular surface could lead to significant asymmetrical stress changes in the bilateral facet joints and ROM instability of the surgical and adjacent segments. These findings suggested that unnecessary and excessive resection should be avoided in PTED to reduce the incidence of low back pain and the risk of postsurgical degeneration.

**Supplementary Information:**

The online version contains supplementary material available at 10.1186/s13018-023-03916-0.

## Introduction

Percutaneous transforaminal endoscopic discectomy (PTED) is a mainstream endoscopic procedure for treating lumbar disk herniation (LDH). The main procedure involves performing a discectomy within the spinal canal through the Kambin triangle, which is a physiologically important channel surrounded by the anterior nerve root, inferior pedicle, and posterior articular facet joint [1]. Advancements in endoscopic surgical devices have expanded the scope of treatment to spinal stenosis [2]. Despite these advancements, multiple risk factors of failure after PTED have been reported [3], which raises the question that if this procedure still requires further evaluation, such as postoperative instability and the degeneration of adjacent segments.

Foraminoplasty is a critical step in PTED. For young adults with a large Kambin triangle, the cannula can be inserted directly into the space of the herniated disk without far dissociation. However, this becomes difficult if there is facet degeneration or a height reduction in the disk, and foraminoplasty is necessary in these situations [4]. The first tool used for laminoplasty was a trephine reamer monitored under fluoroscopy. Later, a trephine drill was developed to reduce nerve root irritation [5]. In recent years, full-endoscopic tools like a visible reamer under endoscopy have improved the laminoplasty procedure [6]. For example, doctors can operate several rounds of resection on the superior articular process (SAP) with significantly less fluoroscopy. With this enhanced capability for bone resection, this procedure can also treat the symptoms of central canal or lateral recess stenosis [7], with the name of transforaminal endoscopic lumbar foraminotomy (TELF) and transforaminal endoscopic lateral recess decompression (TE-LRD) reported in previous studies [8]. However, the partial resection on the inferior articular process (IAP) and isthmus might damage the articular cartilage and capsule and decrease the stability of the target segment, leading to a higher incidence of adjacent segment degeneration (ASD) and severe low back pain (LBP) [9]. Hence, the relationship between the volume of bone removal and the subsequent postoperative degeneration should be considered to further develop of this technique.

Digital simulation technology and finite element analysis (FEA) have become widely used in spinal surgery, such that the segmental spinal units or L1-S1 multi-segmental spinal complexes can be simulated with or without internal fixations [10, 11]. This method assesses the segmental range of motion (ROM), disk and facet stress, and the resultant changes after various spinal surgeries. Matsukawa et al. conducted FEA for screw pullout force in spondylolisthesis [12], while others have also used FEA to analyze local stress following PTED. Most reported finite element models (FEM) were limited to a single-level lumbar unit with simulated conditions that are difficult to quantitatively standardize [13]. However, there is a lack of understanding of the simulating postsurgical changes after the application of the visible trephine. Therefore, this study aimed to construct a multi-level lumbar model of PTED surgery using FEA, mimicking the extents of foraminoplasty with a visible trephine. By assessing the stress on the vertebral body, disk, and facet joints, as well as ROM at different levels during bending and rotational movements, our results could guide the future development of foraminoplasty procedures and postoperative rehabilitation treatments for PTED.

## Methods

A 35-year-old healthy male volunteer, with no history of lumbar spine diseases, trauma, or surgery, was recruited for this study after obtaining approval from the ethics committee and signing the informed consent form. X-ray films of the lumbar spine were taken in the anterior–posterior and lateral positions, as well as the double oblique and dynamic positions to exclude lumbar spine deformity, fracture, and instability. Lumbar CT scans were then performed using a 64-slice GE Lightspeed spiral CT machine with a slice thickness of 0.625 mm, which allowed for the acquisition and export of L3-S1 lumbar CT data in DICOM format from normal adults.

The data were imported into the Mimic 21.0 software (Materialize, Leuven, Belgium) for three-dimensional reconstruction, where different views in coronal, horizontal, and sagittal planes were viewed and the shadow of soft tissue was removed. The editing tool was used to define the image outline and isolate the cross-sectional image via a region-growing process in the target region. Relevant commands, such as repair and erase, were used to obtain the final outline of the model. The Calculate 3D function was then used to generate the triangular geometric model, which was automatically triangulated using the Remesh module. The triangular surface mesh was then smoothed using the STL Smoothener, and the number of error triangles was counted using the Reduced with Quality operation command. Triangle surfaces that did not meet the quality requirements were deleted, and a more accurate STL geometric model of triangular surfaces with an approximate geometric shape, smooth surfaces, and high-quality triangular surface meshes was obtained.

The L3-S1 lumbar vertebra model obtained by three-dimensional reconstruction was a triangular patch model with a rough surface, deformity, distortion, and other issues. The STL file was imported into the Geomagic Studio 2014 software (Geomagic Inc., North Carolina, USA) for surface fitting and smoothening. Triangular patch subdivision, noise reduction, and smoothing processing were performed with the data exported by Mimics21.0. The accurate surface function was used for surface modification to develop a three-dimensional solid geometric model of the L3-S1 segment normal lumbar vertebra.

Further model processing was performed to mimic different degrees of foraminoplasty. When applying the visible trephine of 3.6/6.3 mm endoscope system in PTED, the first resection would remove the ventral part of the L5 SAP apex. If further facetectomy is needed, the second resection would remove the remaining part of the L5 SAP apex. If the procedure aimed to solve stenosis of the central canal, the third resection would remove part of L4 IAP and isthmus. If the procedure aimed to solve stenosis of lateral recess, the fourth resection remove part of L5 pedicle notch to enlarge the left lateral recess. To mimic this surgical process, the study groups were defined as follows:

Group A: The normal group. The model was the intact model of L3-S1 segments.

Group B: The ventral resection group. The ventral side of the left L5 SAP was resected with the articular surface unexposed and intact.

Group C: The apex resection group. The apex of the left L5 SAP was excised with a small area of the articular surface exposed. The capsular ligament in the corresponding area was also removed.

Group D: The ventral + apex + isthmus resection group. The ventral part and apex part of the left L5 SAP were both excised with about 1/3 of the articular surface exposed. A small amount of bone of the left L4 IAP and isthmus was also removed together with the capsular ligament in the corresponding area.

**Fig. 1:**
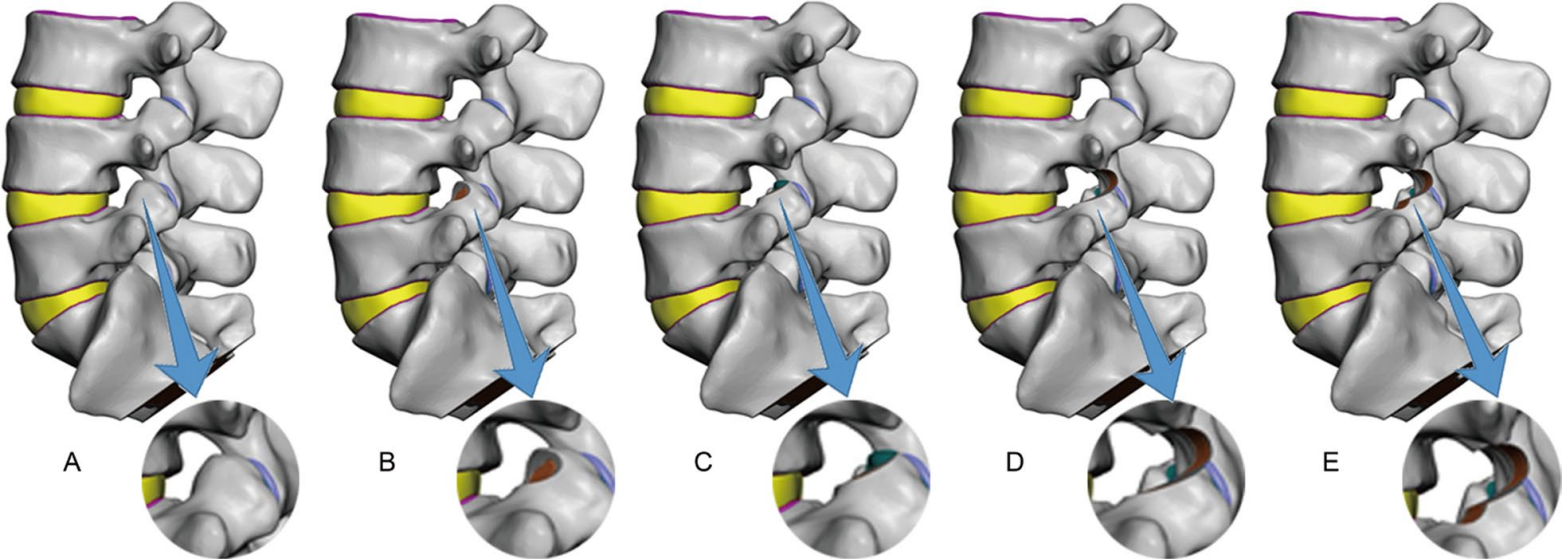
Solid geometric model obtained in Geomagic Studio 2014 for five groups. Group **A**: The normal group with intact model of L3-S1 segments; Group **B**: The ventral resection group. The ventral side of the left L5 SAP was resected with the articular surface unexposed and intact; Group **C**: The apex resection group. The apex of the left L5 SAP was excised with a small area of the articular surface exposed; Group **D**: The ventral + apex + isthmus resection group. The ventral part and apex part of the left L5 SAP were both excised with about 1/3 of the articular surface exposed. A small amount of bone of the left L4 IAP and isthmus was also removed; Group **E**: The SAP + isthmus + lateral recess resection group. From the model of Group **D**, part of the left L5 pedicle notch was removed to enlarge the left lateral recess

Group E: The SAP + isthmus + lateral recess resection group. From the model of Group D, part of the left L5 pedicle notch was removed to enlarge the left lateral recess and the capsular ligament in the corresponding area. The differences of model in Group A–E are displayed in Fig. 1.

After performing a reverse process, ligament models were added to the L3-S1 lumbar spine model, including the anterior longitudinal ligament, posterior longitudinal ligament, capsular ligament, ligamentum flavum, interspinous ligament, supraspinous ligament, intertransverse ligament, and structures such as the cartilage of facet joints, intervertebral disk, and the upper and lower endplates. These models were imported into the MSC.Patran2019 software for structural assembly. The corresponding geometric models of different groups in STP file format were imported into the Hypermesh 14.0 software (Altair Engineering, Troy, Michigan, USA) to generate the mesh. The cortical bone, cancellous bone, intervertebral joint, intervertebral disk nucleus pulposus, annulus fibrosus, and endplate were all meshed with solid elements. To improve the calculation accuracy, convergence, and efficiency, the intervertebral disks (nucleus pulposus and annulus fibrosus) were divided into hexahedral mesh elements (IsoMesh Hex8 element). Other structures were divided into tetrahedral mesh elements (TetMesh Tet4 element), and ligaments were customized as nonlinear spring elements for tension only.

**Fig. 2:**
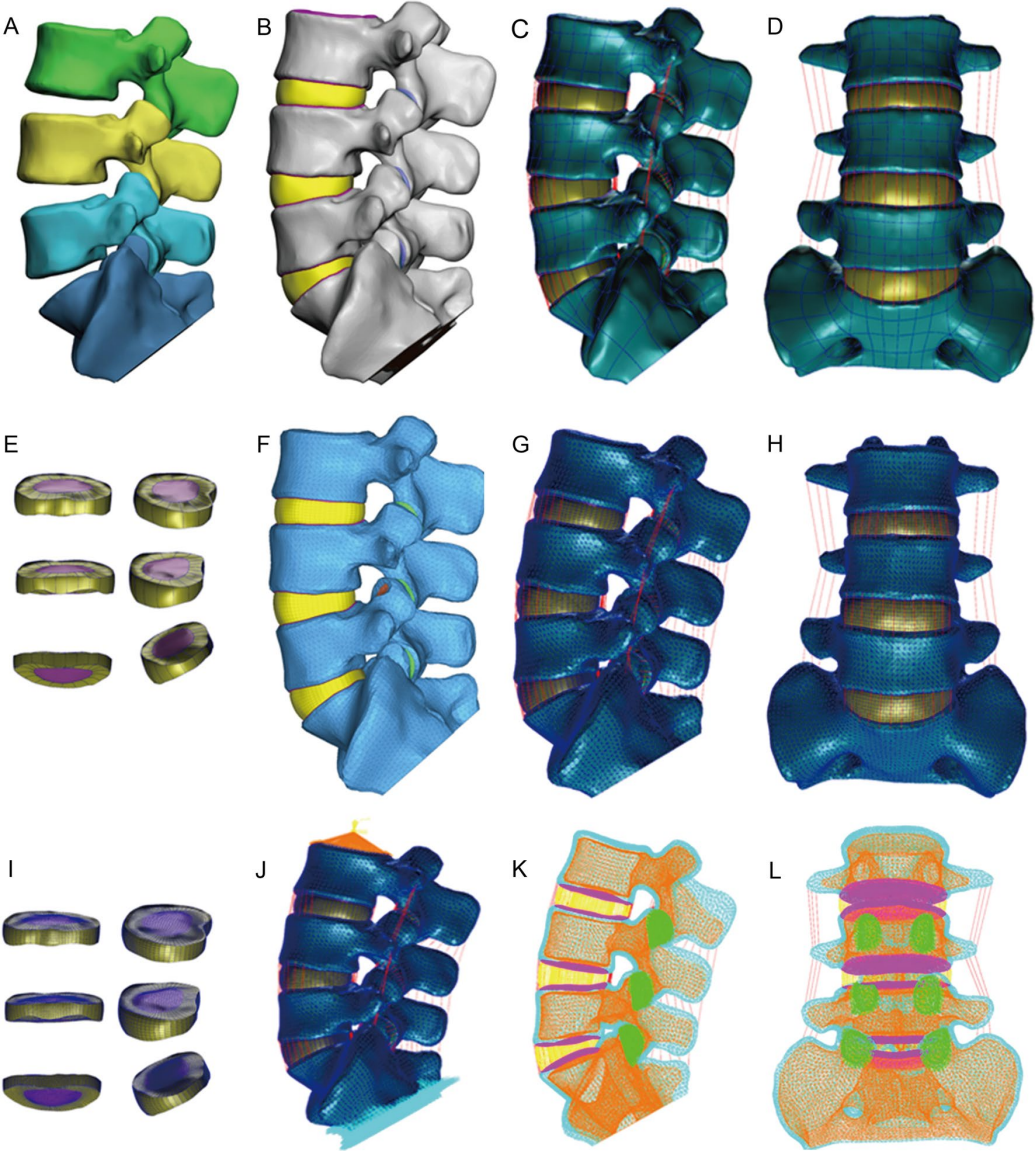
The establishment process of FEA models in different groups. **A** The L3-S1 lumbar vertebra model obtained by 3-dimensional reconstruction in Mimic 21.0; **B** three-dimensional solid geometric model of Group A obtained in Geomagic Studio 2014; **C**, **D**, **E** models were assembled with ligament and intervertebral disk parts in MSC.Patran 2019; **F** models were imported into Hypermesh 14.0 to generate mesh; **G**, **H**, **I**, **J**, **K**, **L** the BDF files were imported into MSC.Patran 2019 for mesh property settings and material parameter definition; and **J** boundary constraints and loads to the model with a vertical load of 500N and a torque of 10N·M were applied to the upper surface of the L3 vertebral body

Next, the BDF files were imported into the finite element pre-processing and processing software, MSC.Patran2019 (Hexagon AB, Stockholm, Sweden). The development process of FEA models is displayed in Fig. 2. The bone structure, upper and lower endplates, facet joints, and intervertebral disk annulus fibrosus were assumed to be isotropic, uniform, and continuous linear elastic materials [10–13]. The nucleus pulposus of the intervertebral disk was set as a hyperelastic material, and the ligaments were customized as nonlinear spring units under tension only. Specific structural material parameters are listed in Tables 1 and 2.

**Table 1:** Material properties for various structural components in the FEA model

Component	Young’s modulus (MPa)	Passion ratio
Cortical bone	12,000	0.30
Cancellous bone	100	0.20
Cartilage endplate	1000	0.40
Posterior structures	3500	0.25
Annulus fiber	450	0.30
Annulus ground	8	0.45
Nucleus pulposus	Hyperelastic C1 = 0.12, C2 = 0.03	
Articular facet joints	10	0.40

**Table 2:** Material properties for various ligamental components in the FEA model

Ligament	Strain (%)	Rigidity (k N/mm)	Strain (%)	Rigidity (k N/mm)	Strain (%)	Rigidity (k N/mm)
ALL	(0,12.2)	347	(12.2,20.3)	787	(20.3, + ∞)	1864
PLL	(0,11.1)	29.5	(11.1,23)	61.7	(23, + ∞)	236
CL	(0,25)	36.0	(25,30)	159	(30, + ∞)	384
ITL	(0,18.2)	0.3	(18.2,23.3)	1.8	(23.3, + ∞)	10.7
LF	(0,5.9)	7.7	(5.9,49)	9.6	(49, + ∞)	58.2
SSL	(0,20)	2.5	(20,25)	5.3	(25, + ∞)	34.0
ISL	(0,20)	1.4	(13.9,20)	1.5	(20, + ∞)	14.7

*ALL* anterior longitudinal ligament, *PLL* posterior longitudinal ligament, *CL* capsular ligament, *ITL* intertransverse ligament, *LF* ligamentum flavum, *SSL* Supraspinal ligament, *ISL* interspinal ligament

Boundary constraints and loads were then applied to the lumbar structural models of each group. The bottom surface nodes of the S1 vertebral body were fixed, restricting six degrees of freedom, while a vertical load of 500N and a torque of 10N·M were applied to the upper surface of the L3 vertebral body to simulate the biomechanical characteristics of the lumbar structure under the motion of flexion, extension, lateral bending, and rotation. The von Mises stress maps of the intervertebral disk, vertebral body, facet joints, and the range of motion (ROM) of the L3-S1 intervertebral disk were then plotted and analyzed.

## Results

### Model validation

**Fig. 3:**
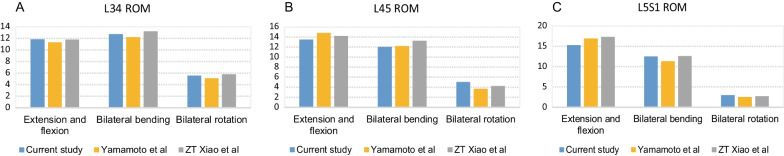
Model validation results compared with previous studies. The results of current study were compared with Yamamoto’s in vitro experimental data [14] and Xiao’s [15] FEA data. The calculated ROM for each segment motion was consistent with the previous studies in terms of both trend and value, thus demonstrating the validity of the normal model utilized in this study. **A** The ROM validation of L34 segment; **B** The ROM validation of L45 segment; **C** The ROM validation of L5S1 segments

To confirm the validity and accuracy of the modeling method, model setting, boundary assumption, and structure simplification used in the finite element simulation analysis process, the same load and constraint conditions were applied to the upper surface of the L3 vertebral body. Biomechanical characteristics of the lumbar structure corresponding to six motion states—flexion, extension, left and right lateral bending, and left and right rotation—were analyzed. Figure 3 displays the ROM for each intervertebral body under different states of motion, and the results were compared with previous in vitro experimental and FEA data [14, 15]. The calculated ROM for each segment motion within this study was consistent with the results of previous studies in terms of both trend and value, thus demonstrating the validity of the normal model in this study.

### Von Mises stresses on L3-S1 vertebral bodies

The impact of various facet resections on the peak stress of the L3-S1 vertebral body was not significantly different (Table 3). In Group A, the peak stress of the L3-S1 vertebral body ranged from about 6.65–10.29 MPa, while Group B reported peak stress of about 6.59–9.88 MPa. Likewise, Group C reported peak stress of about 6.56–9.94 MPa. Corresponding to Group D, the peak stress of the L3-S1 vertebral body was about 6.48–9.99 MPa, and Group E reported about 6.69–9.95 MPa of stress. Although the stress of the vertebral body was elevated in the flexion motion, the changes of peak stress on the vertebral body in each group were not significant in the same motion state, and the range of difference was within 9%.

**Table 3:** The peak stress of L3-S1 vertebral body of five groups

	Group A	Group B	Group C	Group D	Group E
Flexion	10.29	9.88	9.94	9.99	9.95
Extension	7.09	6.47	6.67	7.29	7.39
Left bending	6.90	6.59	6.56	6.67	6.69
Right bending	7.05	6.92	7.02	6.99	7.12
Left rotation	7.47	7.46	6.96	7.13	7.92
Right rotation	6.65	6.67	6.56	6.48	7.14

Group A: The normal group; Group B: The ventral resection group; Group C: The apex resection group; Group D: The ventral + apex + isthmus resection group; and Group E: The SAP + isthmus + lateral recess resection group. The changes of peak stress on the vertebral body for each group was not significant in the same motion state with a range of difference within 9%

### Von Mises stresses on L3-S1 vertebral disks

**Fig. 4:**
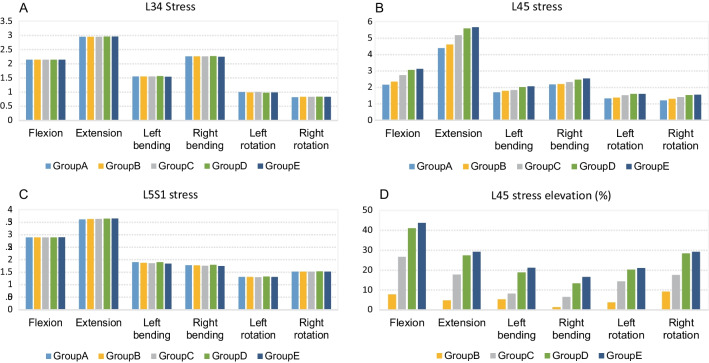
Von Mises stresses on L3-S1 intervertebral disks. **A** and **C** No significant differences were observed in stress changes between groups for the L3/4 and L5/S1 intervertebral disks, with variations within 4%. **B** and **D** Significant stress differences were noted for the L4/5 intervertebral disk across groups, with an increase up to 41% and 44% for Groups D and E in flexion and right rotation motions

There were no significant differences observed in stress changes between the L3/4 and L5/S1 intervertebral disks (Fig. 4). Peak stresses for the L3/4 intervertebral disk ranged between 0.82 and 2.96 MPa, while peak stresses for the L5/S1 intervertebral disk ranged between 1.30 and 3.65 MPa for different motion states. In contrast, significant stress differences were noted in the L4/5 intervertebral disk in each group. As compared to the normal group, Group B experienced an increase in L4/5 stress of less than 9%, whereas Group C reported an increase ranging from 6 to 26% for different motions. In Groups D and E, the bone removal procedure had a greater impact on L4/5 disk stress in various motion states, with an increase of 13–41% and 16–44%, respectively. Among the different motion states, forward flexion resulted in the highest increase in stress, while right rotation had the least impact on stress changes.

### Von Mises stress on L3-S1 bilateral facet joints

The peak stress of the L3/4 facet joints ranged from approximately 0.70–2.44 MPa, while the peak stress of the L4/5 facet joints ranged from approximately 0.64–2.45 MPa. Likewise the peak stress of the L5/S1 facet joints ranged from approximately 0.80–2.50 MPa. Differences among the groups were observed under different motion states. As compared to the stress of the normal group, the stress of the L3/4 and L5/S1 facet joints decreased in most motion states with varying magnitudes, whereas the stress of the L4/5 facet joint increased in most motion states.

Subsequently, the bilateral facet joint data for each spinal segment were extracted and are analyzed separately in Table 4. For the stress of the L3/4 facet joints, a significant decrease was observed during extension and bilateral bending motions. When comparing both sides, the stress on the right facet joint of each group was significantly lower than on the left facet joint, especially during left and right rotation motions.

**Table 4:** Von Mises stress on L3-S1 bilateral facet joints

L34 left FJ	Group A	Group B	Group C	Group D	Group E	L34 right FJ	Group A	Group B	Group C	Group D	Group E
Flexion	0.84	0.82	0.81	0.66	0.66	Flexion	0.93	0.98	0.74	0.76	0.70
Extension	2.17	2.02	1.63	1.43	1.30	Extension	2.44	2.43	1.75	1.60	1.45
Left bending	1.74	1.76	1.38	1.60	1.18	Left bending	1.73	1.57	1.37	1.30	1.03
Right bending	2.15	2.43	1.86	2.04	1.56	Right bending	1.78	1.52	1.42	1.50	1.11
Left rotation	1.31	1.38	1.11	1.30	1.33	Left rotation	1.36	1.18	1.07	1.10	1.09
Right rotation	0.97	1.00	0.82	1.01	0.93	Right rotation	1.07	0.99	0.94	0.89	0.78

L45 left FJ	Group A	Group B	Group C	Group D	Group E	L45 right FJ	Group A	Group B	Group C	Group D	Group E
Flexion	0.64	0.72	0.67	0.67	0.64	Flexion	0.74	0.65	0.65	0.59	0.59
Extension	2.08	1.84	1.81	1.70	1.71	Extension	2.31	2.39	2.36	2.45	2.42
Left bending	1.57	1.25	1.25	1.27	1.23	Left bending	1.59	1.70	1.68	1.59	1.42
Right bending	1.92	1.51	1.56	1.36	1.29	Right bending	1.49	1.82	1.66	1.57	1.63
Left rotation	1.58	1.55	1.52	1.47	1.48	Left rotation	1.21	1.57	1.51	1.72	1.56
Right rotation	1.48	1.23	1.29	1.08	0.88	Right rotation	1.35	1.68	1.61	2.03	2.06

L5S1 left FJ	Group A	Group B	Group C	Group D	Group E	L5S1 right FJ	Group A	Group B	Group C	Group D	Group E
Flexion	1.85	1.79	1.71	1.71	1.76	Flexion	2.42	2.43	2.34	2.50	2.41
Extension	0.73	0.73	0.73	0.73	0.72	Extension	0.81	0.81	0.80	0.82	0.80
Left bending	1.56	1.42	1.48	1.36	1.45	Left bending	1.06	1.13	1.10	1.18	1.20
Right bending	1.47	1.34	1.22	1.30	1.17	Right bending	1.57	1.62	1.48	1.63	1.55
Left rotation	1.21	1.34	1.18	1.30	1.26	Left rotation	2.10	2.10	2.07	2.11	2.11
Right rotation	1.72	1.67	1.73	1.64	1.73	Right rotation	0.97	1.03	1.02	1.04	0.98

The peak stress of the L3/4 facet joints ranged from approximately 0.70–2.44 MPa, the peak stress of the L4/5 facet joints ranged from approximately 0.64–2.45 MPa, and the peak stress of the L5/S1 facet joints ranged from approximately 0.80–2.50 MPa. The results indicated inconsistency stress changes of bilateral facet joints among groups, particularly during bilateral rotation movements

For the stress of the L4/5 facet joints, the stress of the left facet (resection side) increased in each group during anterior flexion motion, with the highest increase observed in Group B. The stress decreased during other motions for each group, with the most prominent reduction occurring during right bending and right rotation motions, especially in Group E. For the L4/5 right facet joint, the stress during anterior flexion in each resection group was lower than in the normal group, especially in Groups D and E. The stress in other motions reported a marked increase after foraminoplasty as compared to the normal group, particularly during bilateral rotational motions in Groups C and D. The stress change pattern of the L4/5 right facet joints demonstrated asymmetric trends as compared to the left facet joints (Fig. 4).

The variation in the L5/S1 facet joint was significantly lesser than in the L3/4 and L4/5 joints. The stress during left rotation motion increased in each resection group and remained almost the same during posterior extension motion. The stress slightly decreased during other motion states for each resection group, particularly during right bending motion in Groups C and E. The stress was slightly elevated on the right facet joint in each resection group, particularly during left bending motion in Groups D and E, with a higher value observed in Group E than in Group D. Nonetheless, the trends stress in the right facet joints was inconsistent with other motions of the right side (Additional File 2: Figures).

### Range of motion (ROM) of L3-S1 intervertebral disks

The ROM of L3-S1 gradually increased from Group A to Group E, especially during flexion, left lateral bending, and right rotation (Table 5). In the normal group, the ROM of L3-S1 ranged from 6.72° to 22.65°. In Group B, the ROM ranged from 6.77° to 22.67°. In Group C, the ROM ranged from 7.36° to 26.42°. The relative motion of L3-S1 ranged from 7.98° to 31.06° in Group D, and 8.05° to 31.67° in Group E. The effect of ventral resection on the relative motion of L3-S1 was found to be insignificant. ROM in Group C reported an increase of 1.1–16.6% in different motions, especially during anterior flexion and right rotation. The changes in ROM were more prominent in Group D (increment of 12.3–37.1%) and Group E (increment of 15.2–39.8%), particularly during anterior flexion, left bending, and right rotation.

**Table 5:** Total ROM of L3-S1 lumbar models in various groups

	Group A	Group B	Group C	Group D	Group E
Flexion	22.65°	22.67°	26.42°	31.06°	31.67°
Extension	17.93°	17.98°	19.93°	21.90°	22.01°
Left bending	18.46°	18.69°	20.11°	22.12°	23.65°
Right bending	18.77°	18.84°	18.97°	21.08°	21.63°
Left rotation	6.72°	6.77°	7.36°	7.98°	8.05°
Right rotation	6.82°	6.83°	7.64°	8.47°	8.76°

The ROM of L3-S1 gradually increased from Group A to Group E, especially during flexion, left lateral bending, and right rotation

**Fig. 5:**
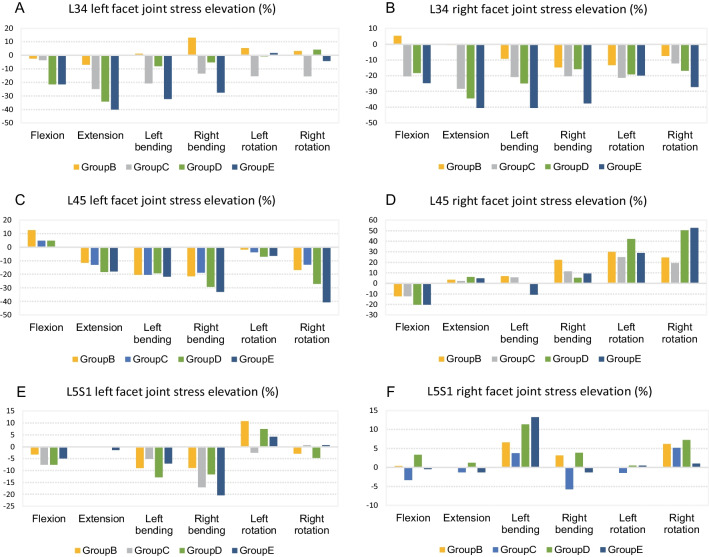
Asymmetric von Mises stress variation on L3-S1 bilateral facet joints. **A**, **B** The stress on the L34 right facet joint of each group was significantly lower than on the left side, especially during left and right rotation motions; **C**, **D** the stress of the L45 left facet joint (resection side) increased in each group during anterior flexion motion, with the highest increase observed in Group B. The stress of L45 right facet joint during anterior flexion in each resection group was lower than in the normal group, especially in Groups and . The stress in other motions showed a marked increase after foraminoplasty compared to the normal group, particularly during bilateral rotational motions in Groups and ; **E**, **F** the stress slightly decreased during other motion states for each resection group, particularly during right bending motion in Groups and . The stress was slightly elevated on the right facet joint in each resection group, particularly during left bending motion in Groups and

The ROM for each disk was analyzed separately (Fig. 5). There was no significant increase in the ROM of the L3/4 disk in Group B. The L3/4 ROM increment in Group C was between − 0.4° and 0.9° (− 0.6 to 14%). A maximum increment in ROM was observed during the flexion movement of Groups D and E, with a maximum of 2.1° (32%). The ROM of Group E (1°, 14.4%) was further increased as compared to Group D (0.5°, 7.82%) during right lateral bending. The increment in ROM for the two groups was less than 1° during other motions.

The ROM of the L4/5 disk reported no noticeable increase in Group B. The increase in Group C was between 0.26° and 1.93°, considerably higher than that in L3/4. The increase in L4/5 for Groups D and E was significantly greater than for Group C, with the highest value in Group E being 19° in the forward flexion motion (55.2%). The elevations in the same motion were similar between the two groups, except for left bending, where the increase in Group E was 1.33° higher than that in Group D (20% of normal volume).

The ROM of the L5/S1 disk did not report a significant change between Group B compared and the normal group. The increment in ROM in Group C ranged from − 0.4° to 0.88° (− 0.63 to 10.54%), which was not significantly different from that observed in the L3/4 disk, but this was significantly lower than that observed in the L4/5 disk. The increase in ROM of the L5/S1 disk in Group E was slightly higher than that in Group D in all motions, with the increase being less than 0.15° (2.16%).

## Discussion

Previous studies have primarily investigated the impact of foraminoplasty on stress changes after PTED [16, 17]. No further evidence was reported since the development of visual trephine and endoscopic dynamic tools. Hence, this study analyzed the overall stress changes and ROMs of the lumbar spine after various degrees of facet resection and further examined the postoperative conditions of the upper and lower adjacent segments. This manuscript provides valuable guidance for intraoperative bone removal and postoperative rehabilitation following PTED.

During the PTED procedure, sheath placement is primarily carried out through Kambin’s triangle. In the early stages of the procedure, Yeung proposed the concept of “inside-out” surgery, which suggested that the cannula should be inserted directly into the intervertebral space at an appropriate angle without removing the bone [18]. However, when a patient has degenerative hyperplasia of the articular process, cannula placement becomes difficult and limits the surgical options. Subsequently, the introduction of trephine and bone drill enabled surgeons to remove the bone from the articular process and expand the Kambin triangle [19]. This resulted in significantly improved surgical flexibility, indications, and safety, as well as better protection of the nerve and complete nerve decompression.

With the development of endoscopic trephine and dynamic systems, surgeons can now remove the bone of the articular process more efficiently, further expanding the scope of PTED [20, 21]. In this study, we analyzed the overall and local stress of the lumbar spine under different bone resection conditions, including the ventral side of the SAP, the apex of the SAP, the isthmus of the L4 lamina, part of the IAP, and the lateral recess/superior notch of the pedicle. This study aimed to cover all possible options for foraminoplasty used in PTED.

Multiple studies have reported the role of the lumbar zygapophysial articular cartilage plays in maintaining the overall stability of the lumbar spine and protecting the annulus fibrosus [22, 23]. Spine surgeons traditionally believed that varying types of facet facetectomy have little impact on postoperative stability [24]. However, Zhou et al. [25] investigated the effect of graded facet resection on cadaveric specimens and found that stability was only compromised when resection exceeded 50%. In our study, we found that ventral facetectomy with minimal bone removal, without compromising the articular surface, had little effect on stability. However, the exposed articular surface after bone removal of the SAP significantly impacted the stability of the lumbar spine. Even a small amount of articular surface exposed by the resection of the SAP apex resulted in an overall increase of approximately 4° (15%) in the L3-S1 ROM and 2° in the L4/5 space. Total resection of the SAP apex, together with isthmic bone removal, resulted in an overall 10° (approximately 35%) increase in the L3-S1 ROM, particularly in flexion, left lateral bending, and right rotation. A 4.19° increase was observed in the L4/5 ROM flexion. These findings were consistent with the findings of Li et al. [26], which reported that ventral facetectomy had little impact on ROM. Exposed articular cartilage significantly increased the ROM under rotational motion, and further resection of the lateral recess bone increased instability. Although this condition did not increase the exposure and destruction of the articular cartilage, it could be related to the decreased bearing capacity of the facet joints and the further loss of integrity of the surrounding important soft tissues such as the joint capsule and the edge of the fibrous ring. These results suggested that the exposure of the articular surface is closely related to the stability after PTED. FEA results could differ from the cadaver results due to elderly and degenerated specimens. Prado et al. [13] further evaluated this factor through finite element analysis and confirmed that the degenerative factor of the intervertebral disk could lead to a decrease in the range of motion of the spine, thus offsetting the effect of facet resection to some extent. Our study also analyzed the ROM changes at each level after angioplasty. Most (> 50%) of the ROM changes came from the surgery segment itself, while the adjacent levels were also affected to a lesser extent. Facet resection could have a long-term impact on both the surgical segment and the lumbar spine as a whole in young patients.

Our study data indicated that several different ranges of foraminoplasty had no significant effect on the peak stress of the L3-S1 vertebral body and were less likely to produce fractures in elderly patients. The difference in the stress of the L3/4 and L5/S1 disks was not significant (less than 4%). However, the stress of L4-5 disks increased significantly after molding, with an increase of less than 9% in Group B and 26% in Group C, while the disk stress of the DE group could increase up to a maximum of 44%. These findings were consistent with those observed by Xie et al. [23] and Li et al. [27], where simple ventral SAP resection had a minimal effect on disk stress, but extended ventral resection or SAP shoulder resection could affect the overall bearing of the facet, thus increasing the disk stress of L45. This increase in disk stress may result in postoperative L45 disk stress concentration, leading to possible recurrence of disk herniation or accelerated space degeneration. However, their reports failed to analyze the disk stress and specific ROMs at adjacent levels. Our study also analyzed disk stress at L3/4 and L5/S1 and confirmed that single-level shaping had little effect on adjacent segments, indicating that single-level PTED had a lower potential for ASD in terms of disk stress.

By extracting and analyzing data related to bilateral facet joints at each level, our study revealed that the stress of L3/4 and L5/S1 facet joints decreased after L4/5 left foraminoplasty. However, the stress of L4/5 facet joints displayed an overall increasing trend. The stress of the right facet of L3/4 decreased more significantly than the left facet, especially during left and right rotation movements. On the other hand, the stress of the left L4/5 facet during flexion movement increased most notably in Group B, whereas the stress during other movements decreased, especially during right lateral bending and right rotation. Furthermore, the stress of the right flexion movement decreased more significantly than normal, especially in Group D and Group E, whereas the stress during other movements increased noticeably, especially in Groups C and D. The stress variation of the L5/S1 articular joints was within 5%, which was much less than that of the upper two spaces. The stress of the left facet increased in left rotation motion and slightly decreased in the other motions of all groups. The stress of the right facet increased significantly in left bending and right rotation in most groups. According to Zeng et al. [16], complete unilateral facet resection led to significant increases in the stress of the contralateral facet, reaching a maximum of 110% during hyperextension. Additionally, axial rotation significantly affected intradiscal pressure. Li et al. [26] observed that the stress of bilateral articular processes increased to varying degrees after L5 facetectomy, with the most notable increase seen during bilateral rotation movements. Our findings were consistent with their results in some aspects, but we provided more detailed information in terms of the specific stress and ROM changes on each disk and facet joints evaluated.

Our study findings suggest that unilateral facet facetectomy can cause significantly inconsistent stress changes in bilateral facet joints, particularly during bilateral rotation movements. This is inconsistent with the current clinical postoperative prevention of hyperextension and hyperflexion movement. Therefore, additional rotation movement protection for patients in the early rehabilitation stages is recommended. Furthermore, the above two studies did not analyze the stress changes of adjacent L3/4 and L5/S1. Our study found inconsistencies in the bilateral facet joint stress of adjacent segments, which may cause long-term effects such as degenerative scoliosis. Hence, it is necessary to further analyze of this difference in stress.

**Fig. 6:**
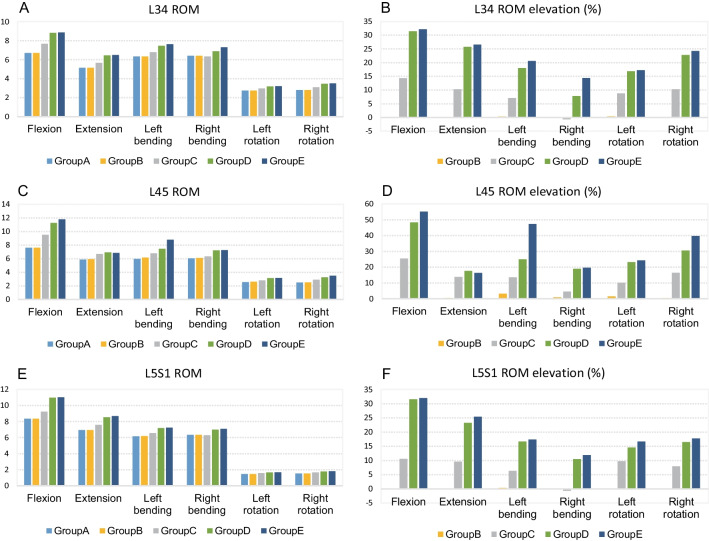
Range and variation of ROM in L3-S1 intervertebral disks. **A**, **B** Maximum increment in ROM appeared during flexion movement of Groups D and E, with a maximum of 2.1° during right bending; **C**, **D** the ROM of L4/5 disk showed no noticeable increase in Group . The increase in L4/5 for Groups and was significantly greater compared to Group **C**, with the highest value in Group E being 19° in forward flexion; **E**, **F** the increment in L5S1 disk was lower than that in L4/5 disk, with values in Group E slightly higher than that in Group D in all motions

Considering our FEA results, we recommend preserving the superior facet bone as much as possible during PTED surgery and avoiding extensive resection. This not only reduces the incidence of postoperative LBP but also lowers the risk of LDH recurrence. Although a full visualization system allows for quicker facet facetectomy, familiarity with bone removal and proficiency in manipulation are still necessary [28]. Otherwise, unexpected enlargement of the facet resection may occur. Future development of the endoscopic dynamic system may enable more precise and controlled foraminoplasty, with the advantages of visualization, accurate bone removal, and safety (reduced endoscopic bleeding caused by bone removal). The only group that fully preserves the facet joints was Group B, with ventral side of the left L5 SAP resected but articular surface unexposed. Results from Table 3 showed that the total ROM of L3-S1 had slight elevation (0.01°–0.23°) compared to normal group. Results from Fig. 6 suggested that the ROM of L34 (0°–0.2°), L45 (0.1°–0.09°) and L5S1 (0°–0.02°) also had slight change compared to normal group. The stress change of vertebral body and disk showed similar results, with value of Group B slightly affected compared to the other treatment groups. The change of flexion, right rotation, and right bending stress in Group B was remarkable compared in other groups. There are several possible reasons for these results. First, the resection in Group B preserved article surface but still damaged some capsular ligament and ligamentum flavum, which affects the stability of soft tissue. Second, the SAP apex remained continuity with SAP body but still lost integrity. During movement, this part of bone had less ability to sustain stress, shifting more stress to the SAP body. Third, the intact of articular surface was only assured in the neutral position. During flexion, right rotation, and right bending movement, the articular surface might still be exposed in this resected area. In the absence of additional internal fixations such as pedicle screws, bone removal of the SAP should still mainly target the ventral region. The removal of the apex of SAP should avoid exposing the dorsal articular surface as much as possible. Lateral recess bone removal should not be a routine procedure for PTED unless lateral recess stenosis is confirmed.

This study had several limitations. Firstly, the stress condition adopted in the present finite element analysis involved a vertical load and torque, which differed significantly from the complex stress conditions in the human body. Though a vertical load can, to some extent, reflect some of the real trends, more relevant conditions for analysis can be applied in future studies. Secondly, this study intended to demonstrate the specific effect of foraminoplasty on postoperative stress changes and stability. Hence, it did not consider the annulus fibrosus damage caused by discectomy, the decrease in nucleus pulposus volume, endplate injury, and other annulus fibrosus factors, and some surgical steps were omitted. The surgical changes of the intervertebral disk were similar between PTED and percutaneous interlaminar endoscopic discectomy (PIED), and previous research findings can be consulted in this regard. The third limitation that we were unable to apply “nerve decompression” effect to the FEA model may contribute to changes in ROM of the spine in clinical practice when the nerve pressure is relieved. Future work on FEA should pay attention to find a solution to this issue.

## Conclusions

In this study, we constructed a multi-segment foraminoplasty model of PTED surgery under visual trephine using the FEA method. Our results indicate that enlarged resection and exposure of the articular surface could lead to significant asymmetrical stress changes in the bilateral facet joints and disk ROM instability of the surgical and adjacent segments. These findings suggested that during PTED surgery, it is crucial to preserve the superior facet as much as possible. Unnecessary and excessive resection should be avoided to reduce the incidence of LBP and the risk of postsurgical degeneration at the surgical and adjacent segments.

## Supplementary Information

**Additional file 1:** Supplementary Figure legends.

**Additional file 2:** **Figure 1.** The von Mises stress nephograms of the vertebral body, intervertebral disc, and facet joints in five groups under six motion. **A1-4:** Group A under flexion motion; **A5-8:** Group A under extension motion; **A9-12:** Group A under left bending motion; **A13-16:** Group A under right bending motion; **A17-20:** Group A under left rotation motion; **A21-24:** Group A under right rotation motion. **B1-4:** Group B under flexion motion. **B5-8:** Group B under extension motion; **B9-12:** Group B under left bending motion; **B13-16:** Group B under right bending motion; **B17-20:** Group B under left rotation motion; **B21-24:** Group B under right rotation motion. **C1-4:** Group C under flexion motion; **C5-8:** Group C under extension motion; **C9-12:** Group C under left bending motion; **C13-16:** Group C under right bending motion; **C17-20:** Group C under left rotation motion; **C21-24:** Group C under right rotation motion. **D1-4:** Group D under flexion motion; **D5-8:** Group D under extension motion; **D9-12:** Group D under left bending motion; **D13-16:** Group D under right bending motion; **D17-20:** Group D under left rotation motion; **D21-24:** Group D under right rotation motion. **E1-4:** Group E under flexion motion; **E5-8:** Group E under extension motion; **E9-12:** Group E under left bending motion; **E13-16:** Group E under right bending motion; **E17-20:** Group E under left rotation motion; **E21-24:** Group E under right rotation motion.

## Data Availability

Not applicable.

## References

[1] Fanous AA, Tumialan LM, Wang MY (2019). Kambin's triangle: definition and new classification schema. J Neurosurg-Spine.

[2] Yang J, Guo C, Kong Q, Zhang B, Wang Y, Zhang L (2020). Learning curve and clinical outcomes of percutaneous endoscopic transforaminal decompression for lumbar spinal stenosis. Int Orthop.

[3] Jitpakdee K, Liu Y, Kim Y, Kotheeranurak V, Kim J (2023). Factors associated with incomplete clinical improvement in patients undergoing transforaminal endoscopic lumbar discectomy for lumbar disc herniation. Eur Spine J.

[4] Yu Y, Zhou Q, Xie YZ, Wang XL, Fan XH, Gu DW (2020). Effect of percutaneous endoscopic lumbar foraminoplasty of different facet joint portions on lumbar biomechanics: a finite element analysis. Orthop Surg.

[5] Wang K, Hong X, Zhou BY, Bao JP, Xie XH, Wang F (2015). Evaluation of transforaminal endoscopic lumbar discectomy in the treatment of lumbar disc herniation. Int Orthop.

[6] Xiong C, Li T, Kang H, Hu H, Han J, Xu F (2019). Early outcomes of 270-degree spinal canal decompression by using TESSYS-ISEE technique in patients with lumbar spinal stenosis combined with disk herniation. Eur Spine J.

[7] Cheng XK, Chen B (2020). Percutaneous transforaminal endoscopic decompression for geriatric patients with central spinal stenosis and degenerative lumbar spondylolisthesis: a novel surgical technique and clinical outcomes. Clin Interv Aging.

[8] Jitpakdee K, Liu Y, Dong H, Kotheeranurak V, Siravich S, Kim J (2023). Minimally invasive endoscopy in spine surgery: Where are we now?. Eur Spine J.

[9] Li J, Zhang X, Xu W, Xi Z, Xie L (2019). Reducing the extent of facetectomy may decrease morbidity in failed back surgery syndrome. BMC Musculoskel Dis.

[10] Lu T, Lu Y (2019). Comparison of biomechanical performance among posterolateral fusion and transforaminal, extreme, and oblique lumbar interbody fusion: a finite element analysis. World Neurosurg.

[11] Lo C, Tsai K, Zhong Z, Chen S, Hung C (2011). Biomechanical differences of Coflex-F and pedicle screw fixation combined with TLIF or ALIF—a finite element study. Comput Method Biomech.

[12] Matsukawa K, Yato Y, Imabayashi H, Hosogane N, Asazuma T, Chiba K (2016). Biomechanical evaluation of lumbar pedicle screws in spondylolytic vertebrae: comparison of fixation strength between the traditional trajectory and a cortical bone trajectory. J Neurosurg-Spine.

[13] Prado M, Mascoli C, Giambini H (2022). Discectomy decreases facet joint distance and increases the instability of the spine: a finite element study. Comput Biol Med.

[14] Yamamoto I, Panjabi MM, Crisco T, Oxland T (1989). Three-dimensional movements of the whole lumbar spine and lumbosacral joint. Spine.

[15] Xiao Z, Wang L, Gong H, Zhu D (2012). Biomechanical evaluation of three surgical scenarios of posterior lumbar interbody fusion by finite element analysis. Biomed Eng Online.

[16] Zeng Z, Zhu R, Wu Y, Zuo W, Yu Y, Wang J (2017). Effect of graded facetectomy on lumbar biomechanics. J Healthc Eng..

[17] Yang J, Chu L, Chen C, Wang X, Xie P, Deng R (2018). Foraminoplasty at the tip or base of the superior articular process for lateral recess stenosis in percutaneous endoscopic lumbar discectomy: a multicenter, retrospective, controlled study with 2-year follow-up. Biomed Res Int.

[18] Tsou PM, Yeung AT (2002). Transforaminal endoscopic decompression for radiculopathy secondary to intracanal noncontained lumbar disc herniations: outcome and technique. Spine J.

[19] Schubert M, Hoogland T (2005). Endoscopic transforaminal nucleotomy with foraminoplasty for lumbar disk herniation. Oper Orthop Traumato.

[20] Ke W, Zhi J, Hua W, Wang B, Lu S, Fan L (2020). Percutaneous posterior full-endoscopic cervical foraminotomy and discectomy: a finite element analysis and radiological assessment. Comput Method Biomech.

[21] Sun F, Liang Q, Yan M, Wang H, Liu Z, Li F (2020). Unilateral laminectomy by endoscopy in central lumbar canal spinal stenosis: technical note and early outcomes. Spine.

[22] Xie Y, Wang X, Jian Q, Fan X, Yu Y, Gu D (2020). Three dimensional finite element analysis used to study the influence of the stress and strain of the operative and adjacent segments through different foraminnoplasty technique in the PELD: Study protocol clinical trial (SPIRIT Compliant). Medicine.

[23] Xie Y, Zhou Q, Wang X, Jian Q, Fan X, Yu Y (2020). The biomechanical effects of foraminoplasty of different areas under lumbar percutaneous endoscopy on intervertebral discs: a 3D finite element analysis. Medicine.

[24] Erbulut DU (2014). Biomechanical effect of graded facetectomy on asymmetrical finite element model of the lumbar spine. Turk Neurosurg.

[25] Zhou Y, Luo G, Chu TW, Wang J, Li CQ, Zheng WJ (2007). The biomechanical change of lumbar unilateral graded facetectomy and strategies of its microsurgical reconstruction: report of 23 cases. Zhonghua Yi Xue Za Zhi.

[26] Li XR, Yu J, Zhang W, Gao GM, Han L, Chen L (2020). Biomechanical model study of the effect of partial facetectomy on lumbar stability under percutaneous endoscopy. World Neurosurg.

[27] Li J, Li H, He Y, Zhang X, Xi Z, Wang G (2020). The protection of superior articular process in percutaneous transforaminal endoscopic discectomy should decreases the risk of adjacent segment diseases biomechanically. J Clin Neurosci.

[28] Lewandrowski KU, Soriano-Sanchez JA, Zhang X, Ramirez LJ, Soriano SS, Rugeles OJ, et al. Surgeon motivation, and obstacles to the implementation of minimally invasive spinal surgery techniques. J Spine Surg. 2020;6(Suppl 1):S249-S259. 10.21037/jss.2019.08.02. 10.21037/jss.2019.08.02 PMC7063314 32195432

