# Supplementary material for: Visible trephine-based foraminoplasty in PTED leads to asymmetrical stress changes and instability in the surgical and adjacent segments: a finite element analysis
Source: J Orthop Surg Res. 2023 Jun 13;18:431. doi: 10.1186/s13018-023-03916-0 (PMC10265803; doi:10.1186/s13018-023-03916-0)
Supplement: Supplementary file 1 — Additional file 1: Supplementary Figure legends. [file 13018_2023_3916_MOESM1_ESM.docx]

**Supplementary Figure legends**

A1-4: The von Mises stress nephograms of the vertebral body, intervertebral disc, and facet joints in Group A under flexion motion.

A5-8: The von Mises stress nephograms of the vertebral body, intervertebral disc, and facet joints in Group A under extension motion.

A9-12: The von Mises stress nephograms of the vertebral body, intervertebral disc, and facet joints in Group A under left bending motion.

A13-16: The von Mises stress nephograms of the vertebral body, intervertebral disc, and facet joints in Group A under right bending motion.

A17-20: The von Mises stress nephograms of the vertebral body, intervertebral disc, and facet joints in Group A under left rotation motion.

A21-24: The von Mises stress nephograms of the vertebral body, intervertebral disc, and facet joints in Group A under right rotation motion.

B1-4: The von Mises stress nephograms of the vertebral body, intervertebral disc, and facet joints in Group B under flexion motion.

B5-8: The von Mises stress nephograms of the vertebral body, intervertebral disc, and facet joints in Group B under extension motion.

B9-12: The von Mises stress nephograms of the vertebral body, intervertebral disc, and facet joints in Group B under left bending motion.

B13-16: The von Mises stress nephograms of the vertebral body, intervertebral disc, and facet joints in Group B under right bending motion.

B17-20: The von Mises stress nephograms of the vertebral body, intervertebral disc, and facet joints in Group B under left rotation motion.

B21-24: The von Mises stress nephograms of the vertebral body, intervertebral disc, and facet joints in Group B under right rotation motion.

C1-4: The von Mises stress nephograms of the vertebral body, intervertebral disc, and facet joints in Group C under flexion motion.

C5-8: The von Mises stress nephograms of the vertebral body, intervertebral disc, and facet joints in Group C under extension motion.

C9-12: The von Mises stress nephograms of the vertebral body, intervertebral disc, and facet joints in Group C under left bending motion.

C13-16: The von Mises stress nephograms of the vertebral body, intervertebral disc, and facet joints in Group C under right bending motion.

C17-20: The von Mises stress nephograms of the vertebral body, intervertebral disc, and facet joints in Group C under left rotation motion.

C21-24: The von Mises stress nephograms of the vertebral body, intervertebral disc, and facet joints in Group C under right rotation motion.

D1-4: The von Mises stress nephograms of the vertebral body, intervertebral disc, and facet joints in Group D under flexion motion.

D5-8: The von Mises stress nephograms of the vertebral body, intervertebral disc, and facet joints in Group D under extension motion.

D9-12: The von Mises stress nephograms of the vertebral body, intervertebral disc, and facet joints in Group D under left bending motion.

D13-16: The von Mises stress nephograms of the vertebral body, intervertebral disc, and facet joints in Group D under right bending motion.

D17-20: The von Mises stress nephograms of the vertebral body, intervertebral disc, and facet joints in Group D under left rotation motion.

D21-24: The von Mises stress nephograms of the vertebral body, intervertebral disc, and facet joints in Group D under right rotation motion.

E1-4: The von Mises stress nephograms of the vertebral body, intervertebral disc, and facet joints in Group E under flexion motion.

E5-8: The von Mises stress nephograms of the vertebral body, intervertebral disc, and facet joints in Group E under extension motion.

E9-12: The von Mises stress nephograms of the vertebral body, intervertebral disc, and facet joints in Group E under left bending motion.

E13-16: The von Mises stress nephograms of the vertebral body, intervertebral disc, and facet joints in Group E under right bending motion.

E17-20: The von Mises stress nephograms of the vertebral body, intervertebral disc, and facet joints in Group E under left rotation motion.

E21-24: The von Mises stress nephograms of the vertebral body, intervertebral disc, and facet joints in Group E under right rotation motion.
