# Supplementary figures and images for: Visible trephine-based foraminoplasty in PTED leads to asymmetrical stress changes and instability in the surgical and adjacent segments: a finite element analysis
Source: J Orthop Surg Res. 2023 Jun 13;18:431. doi: 10.1186/s13018-023-03916-0 (PMC10265803; doi:10.1186/s13018-023-03916-0)

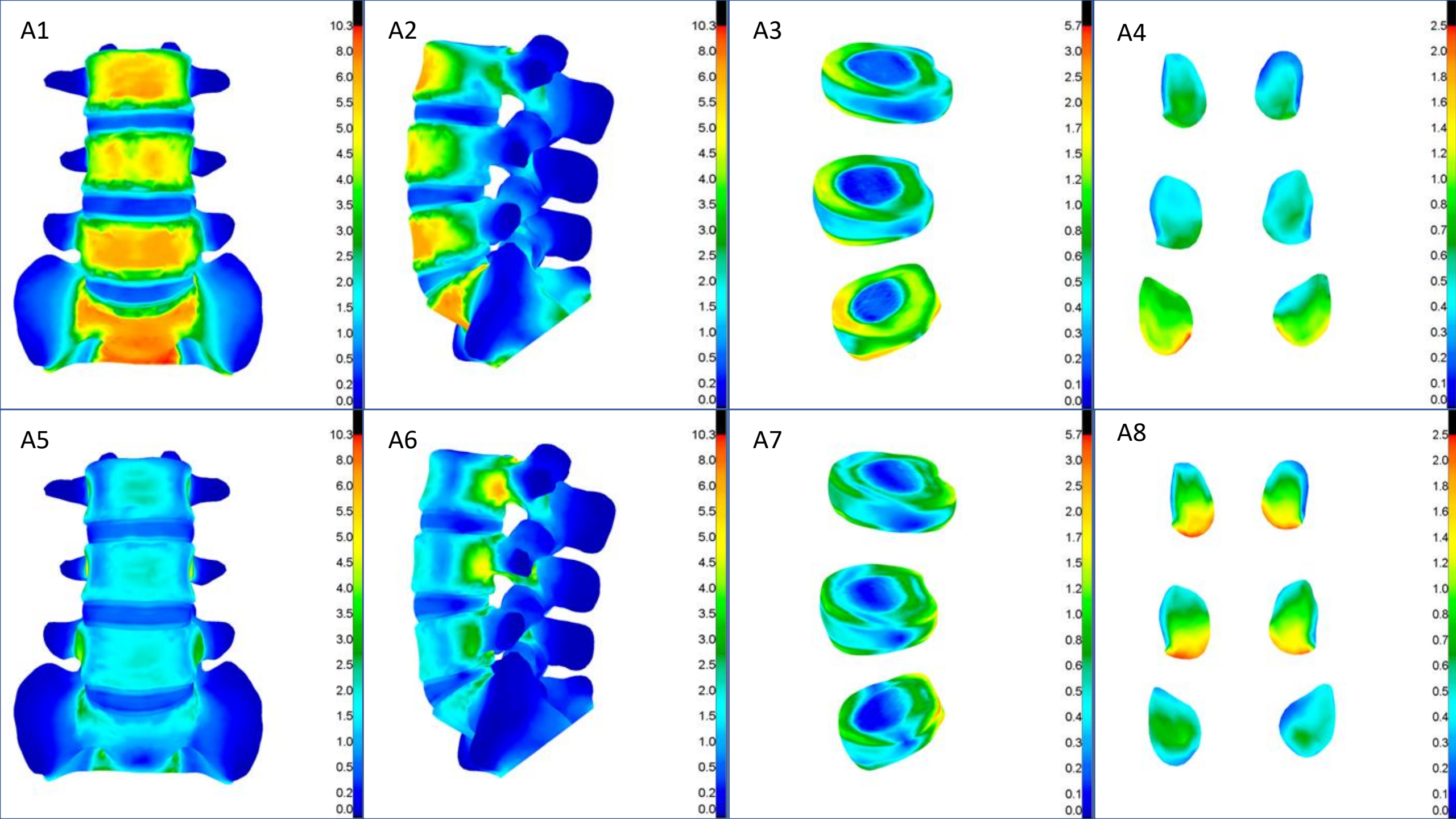

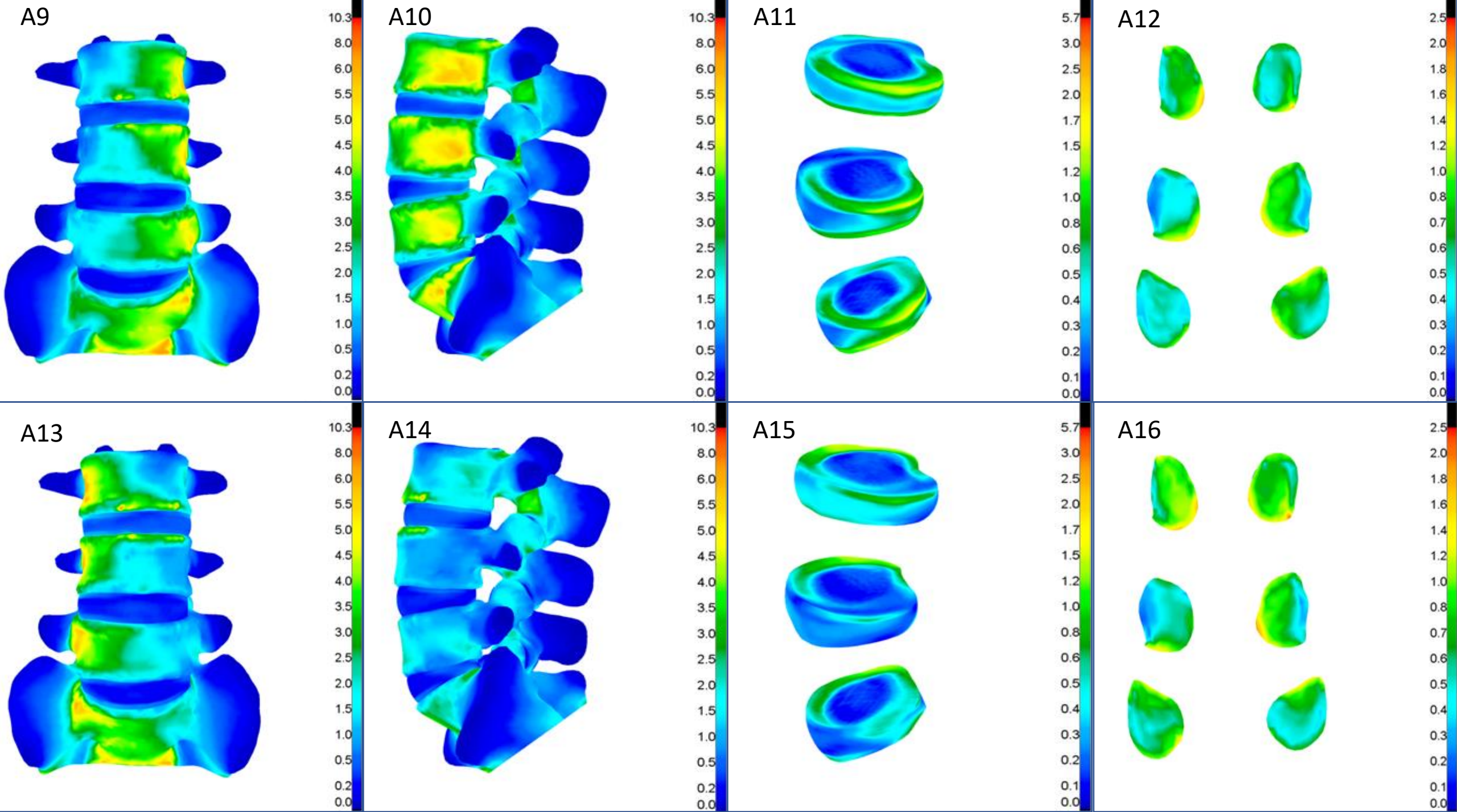

A17

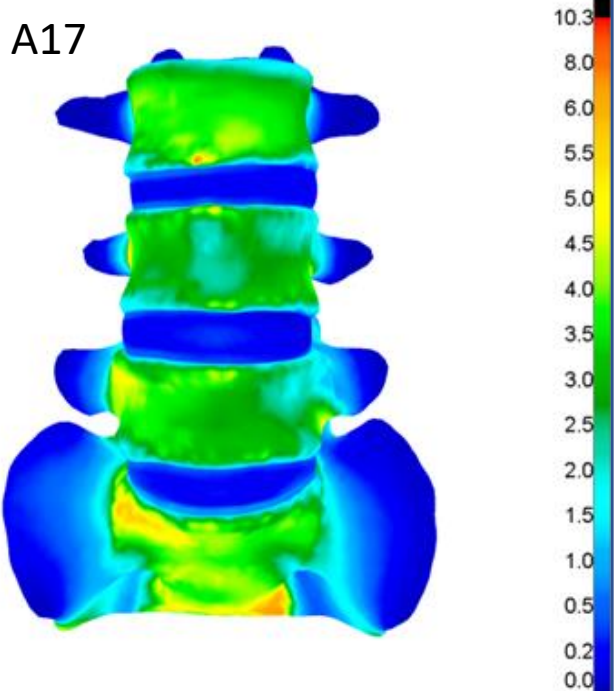

A18

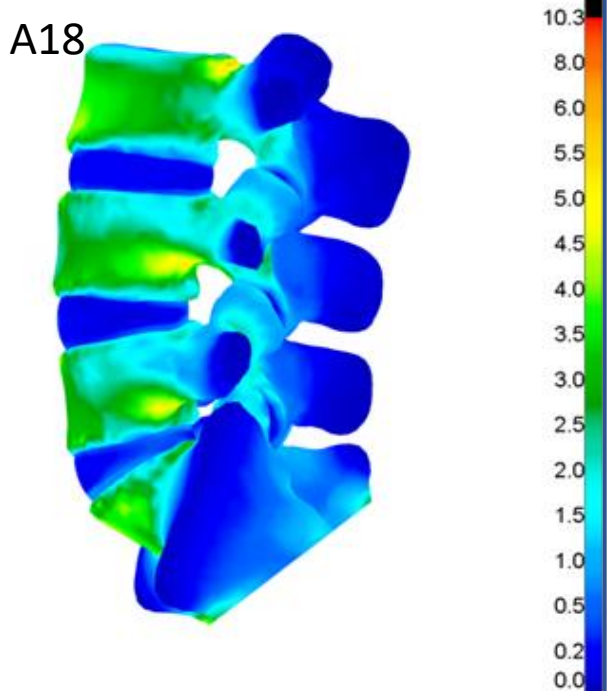

A19

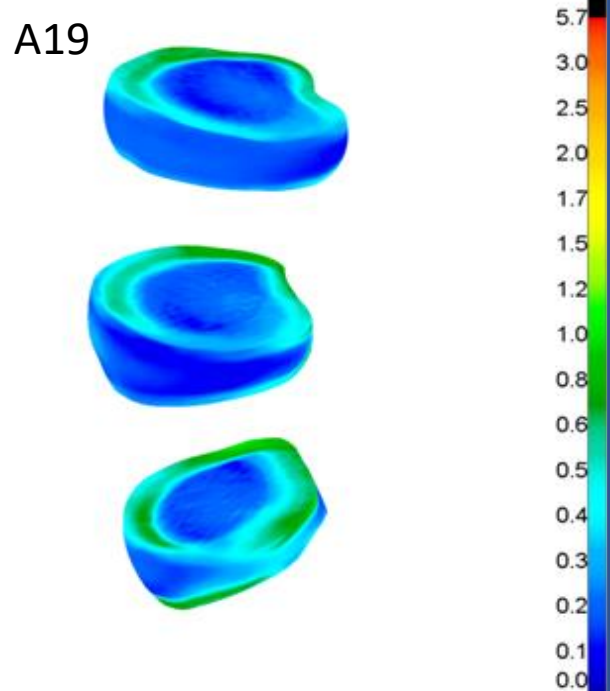

A20

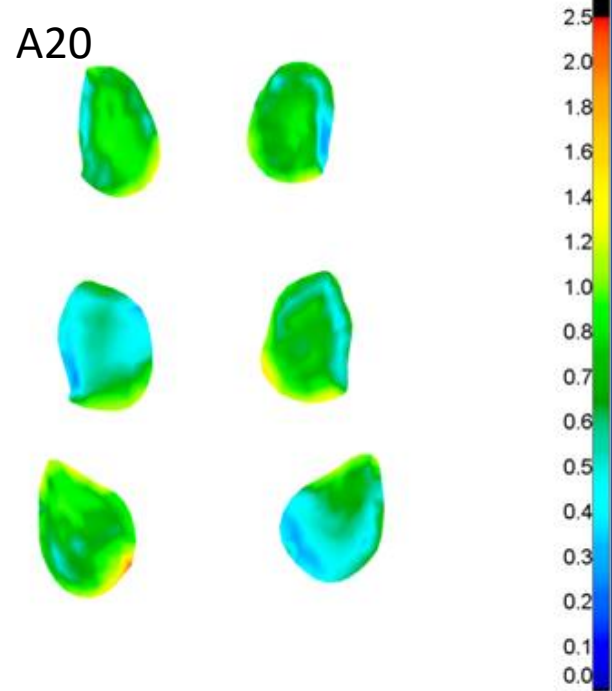

A21

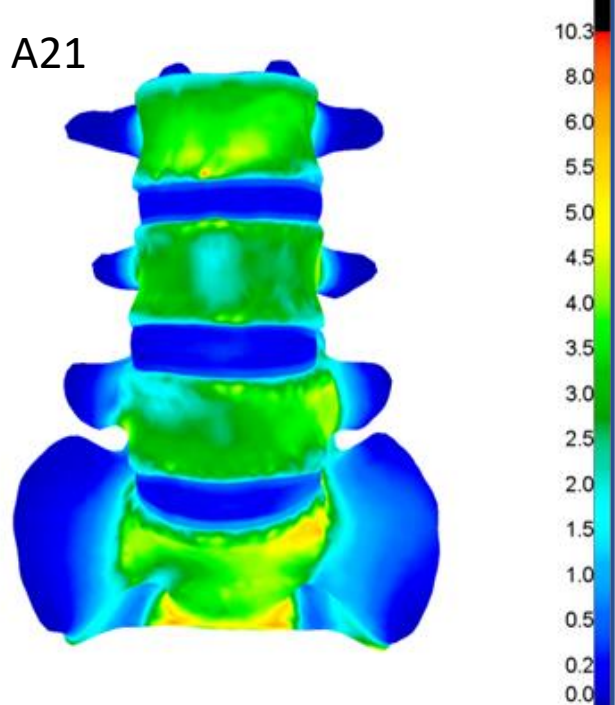

A22

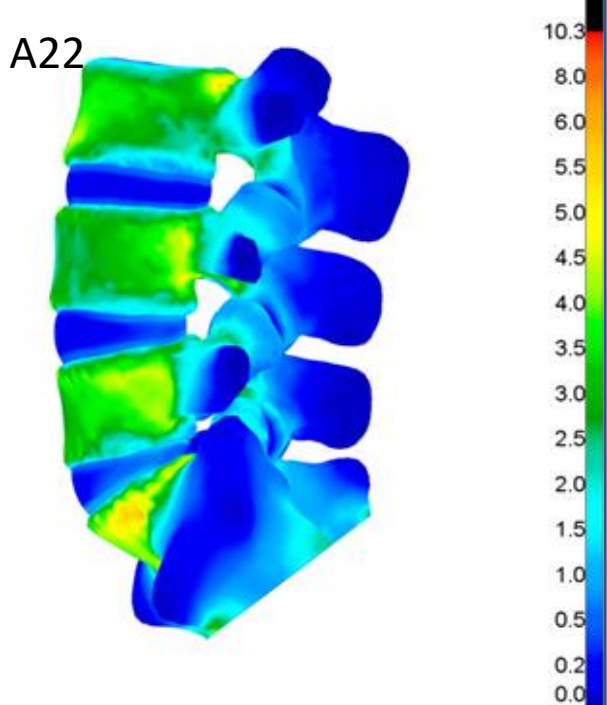

A23

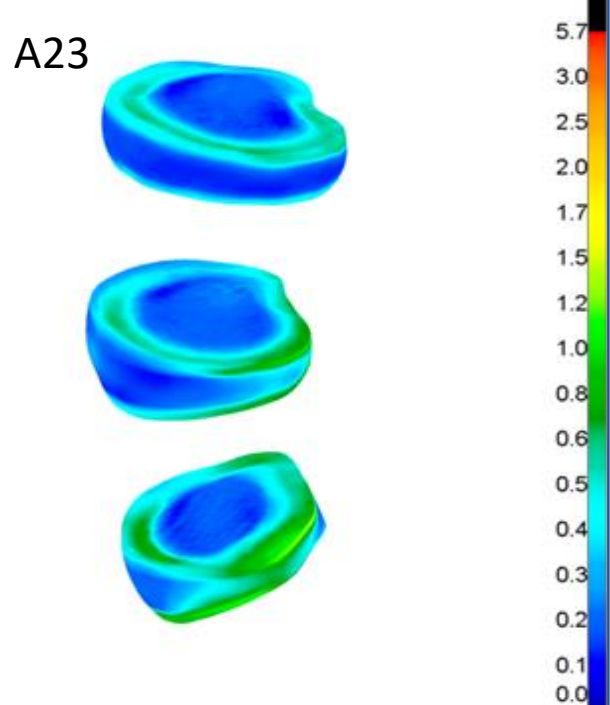

A24

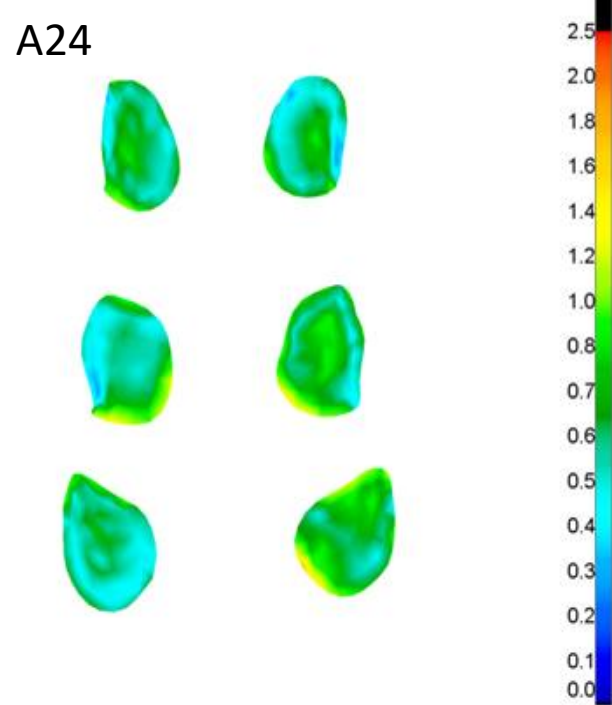

B1

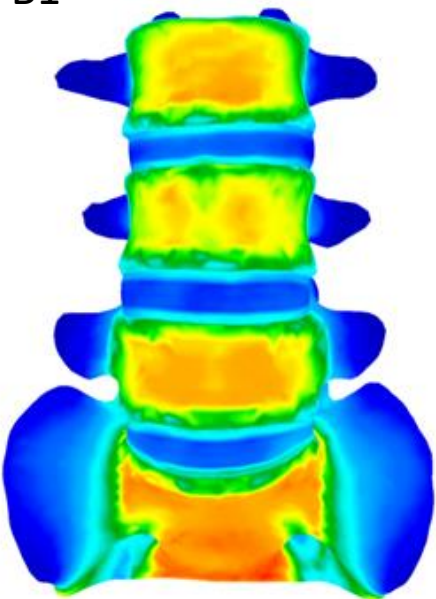

B2

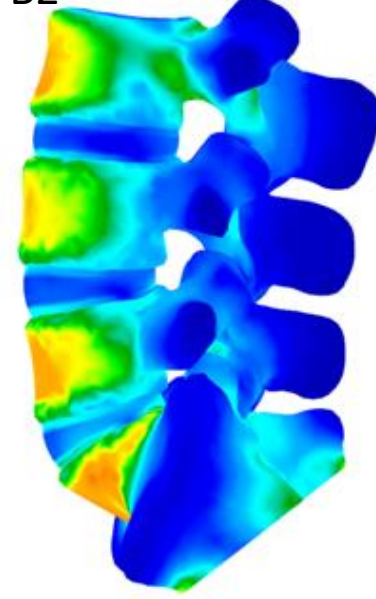

B3

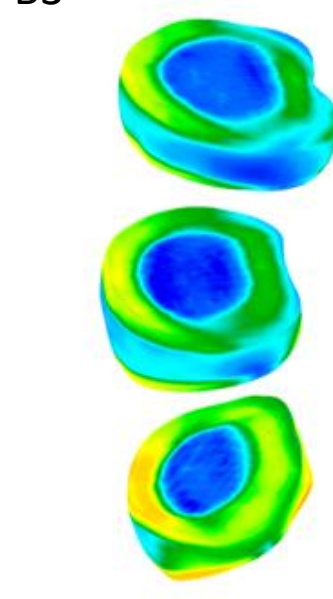

B4

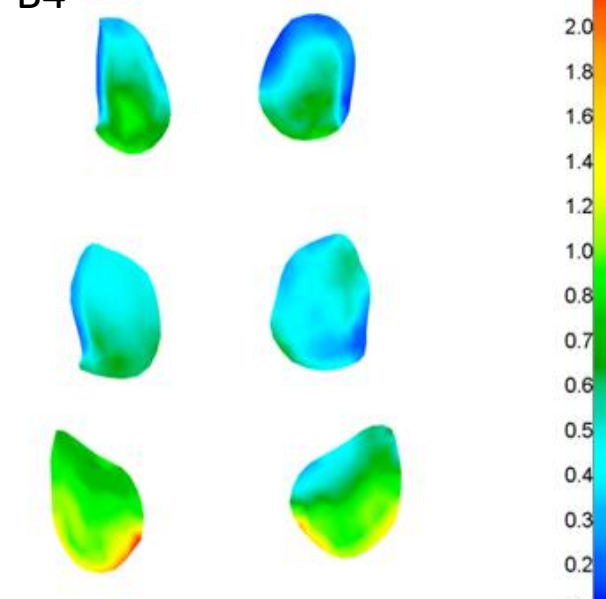

B5

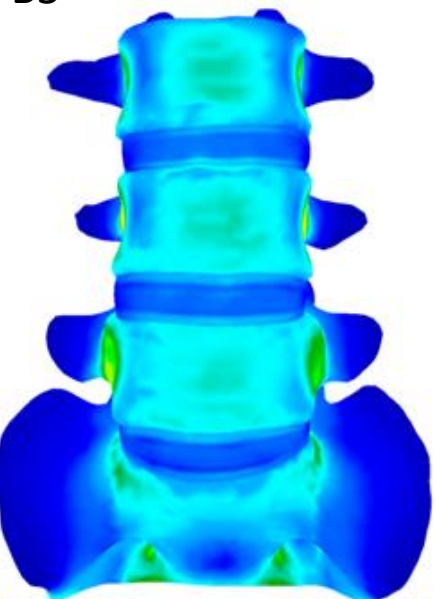

B6

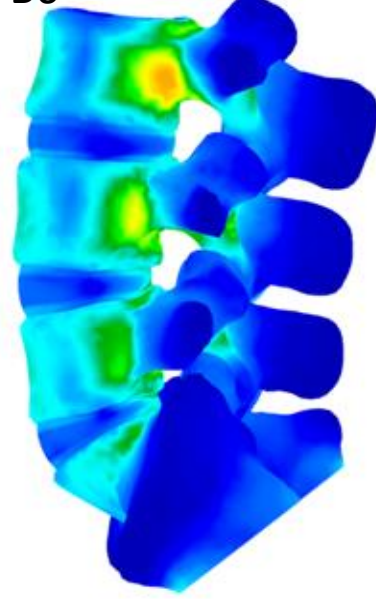

B7

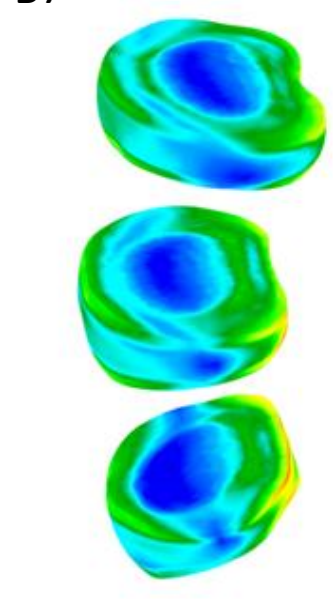

B8

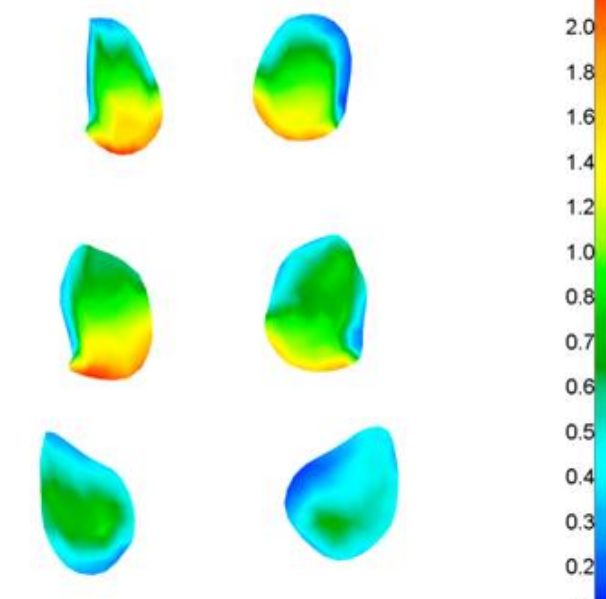

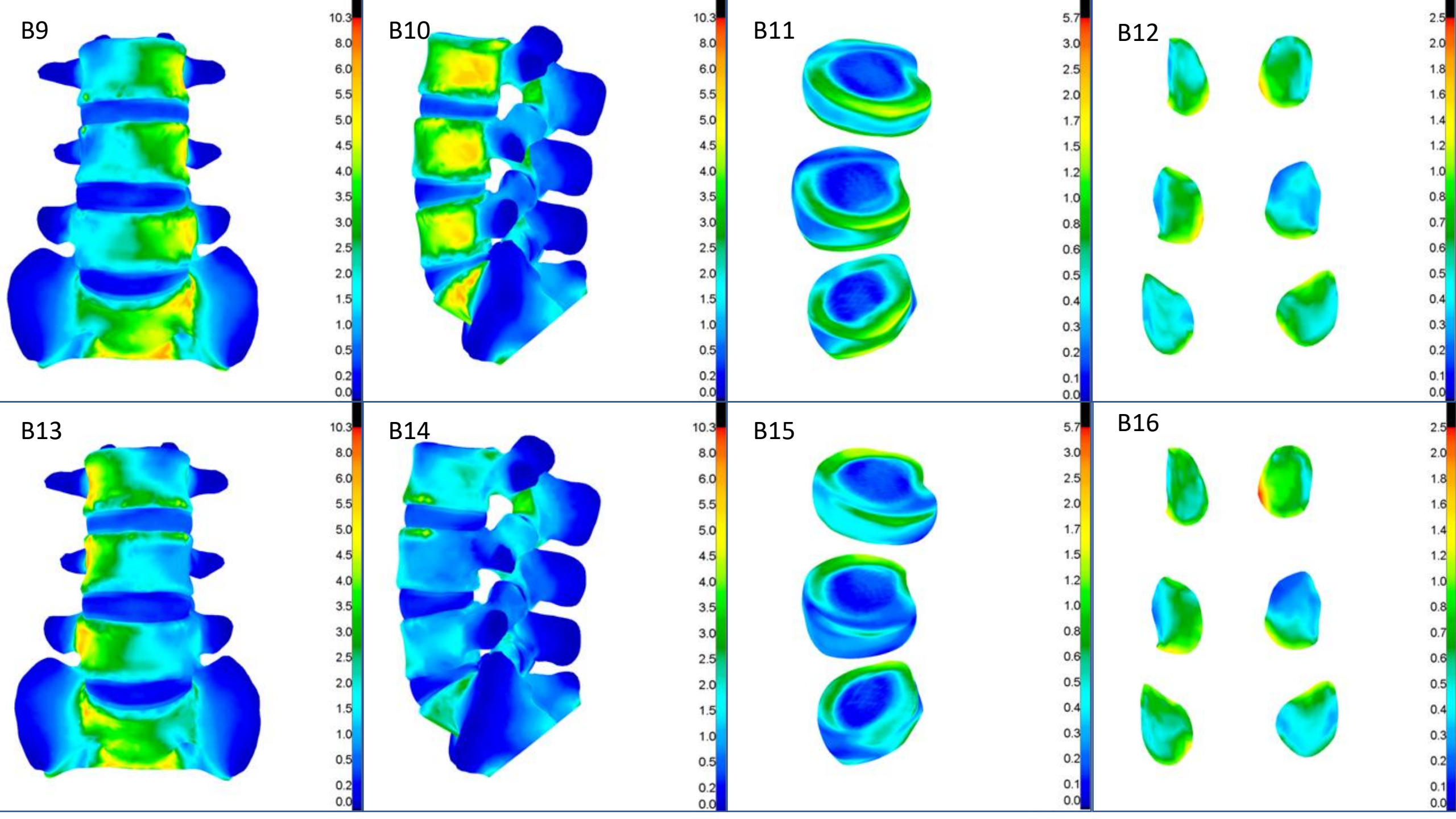

B17

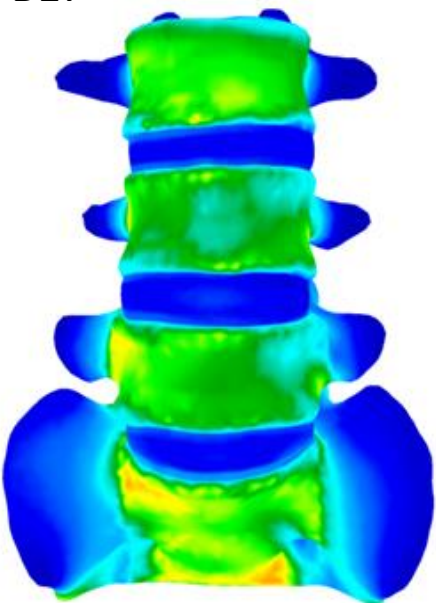10.3  
8.0  
6.0  
5.5  
5.0  
4.5  
4.0  
3.5  
3.0  
2.5  
2.0  
1.5  
1.0  
0.5  
0.2  
0.0

B18

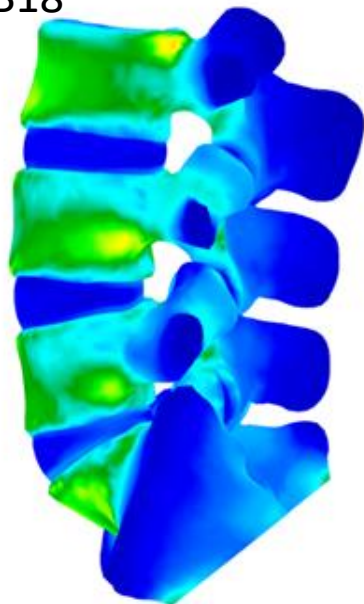10.3  
8.0  
6.0  
5.5  
5.0  
4.5  
4.0  
3.5  
3.0  
2.5  
2.0  
1.5  
1.0  
0.5  
0.2  
0.0

B19

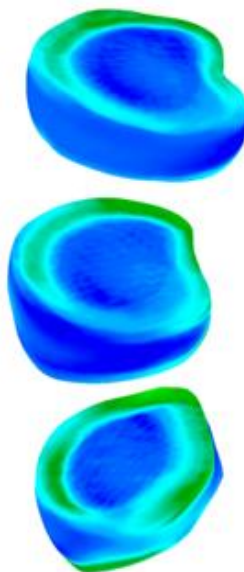5.7  
3.0  
2.5  
2.0  
1.7  
1.5  
1.2  
1.0  
0.8  
0.6  
0.5  
0.4  
0.3  
0.2  
0.1  
0.0

B20

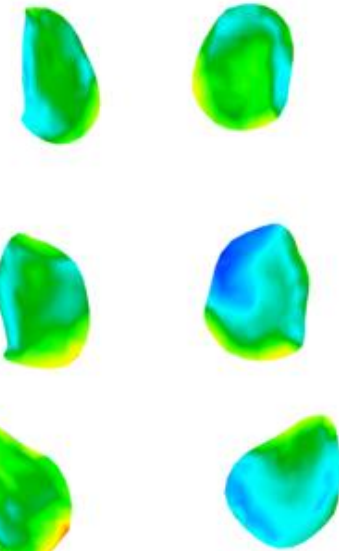2.5  
2.0  
1.8  
1.6  
1.4  
1.2  
1.0  
0.8  
0.7  
0.6  
0.5  
0.4  
0.3  
0.2  
0.1  
0.0

B21

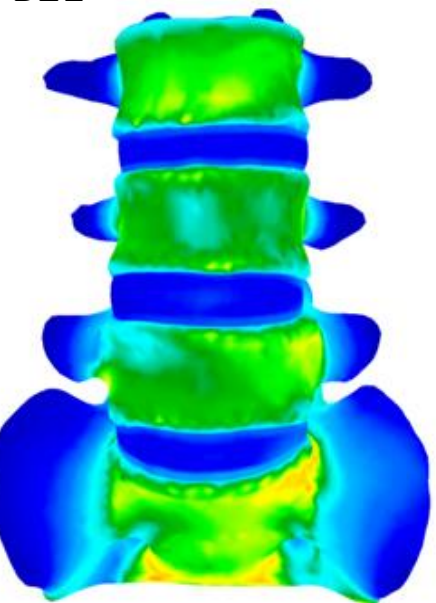10.3  
8.0  
6.0  
5.5  
5.0  
4.5  
4.0  
3.5  
3.0  
2.5  
2.0  
1.5  
1.0  
0.5  
0.2  
0.0

B22

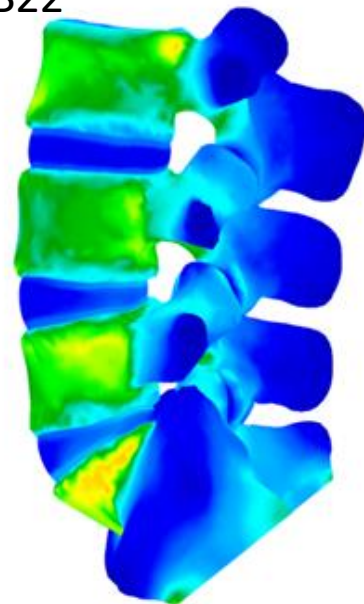10.3  
8.0  
6.0  
5.5  
5.0  
4.5  
4.0  
3.5  
3.0  
2.5  
2.0  
1.5  
1.0  
0.5  
0.2  
0.0

B23

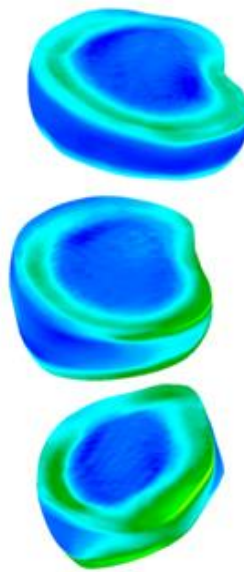5.7  
3.0  
2.5  
2.0  
1.7  
1.5  
1.2  
1.0  
0.8  
0.6  
0.5  
0.4  
0.3  
0.2  
0.1  
0.0

B24

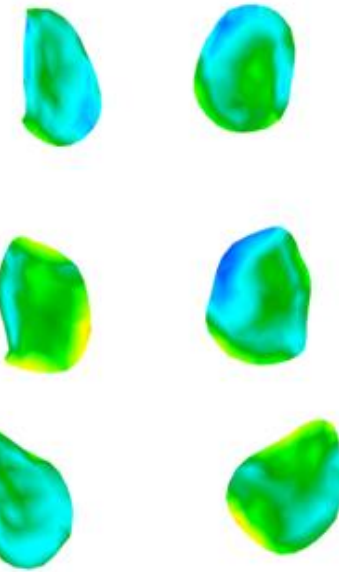2.5  
2.0  
1.8  
1.6  
1.4  
1.2  
1.0  
0.8  
0.7  
0.6  
0.5  
0.4  
0.3  
0.2  
0.1  
0.0

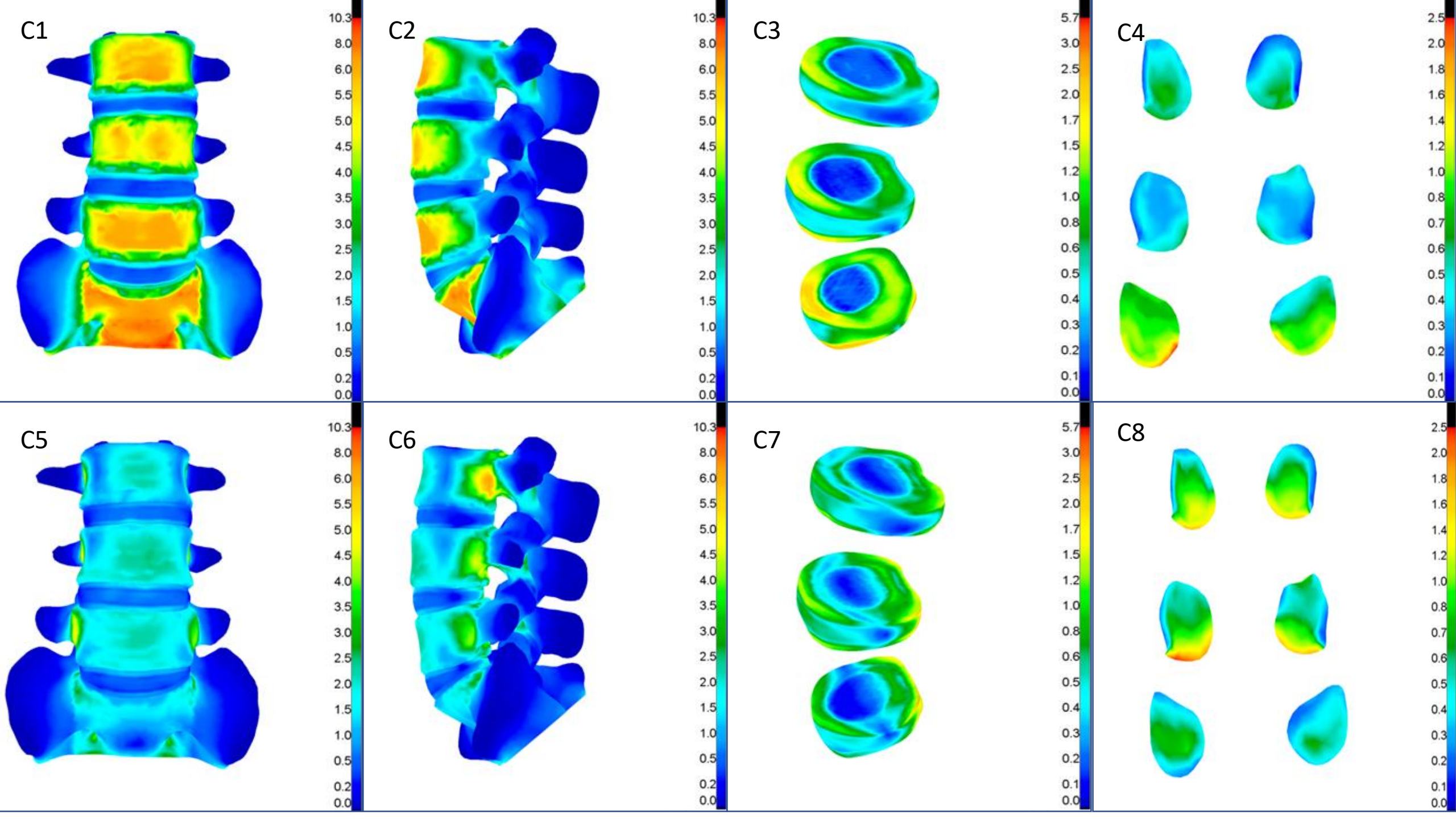

C9

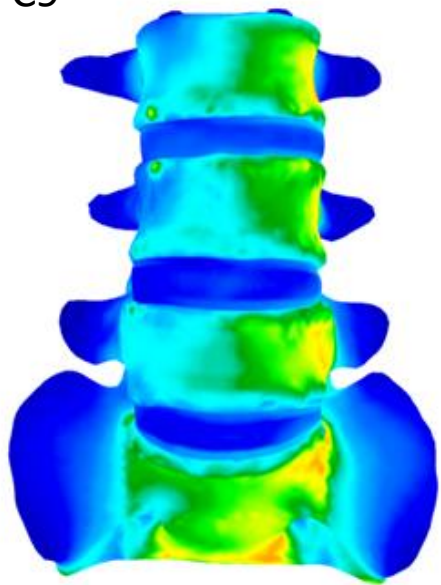10.3  
8.0  
6.0  
5.5  
5.0  
4.5  
4.0  
3.5  
3.0  
2.5  
2.0  
1.5  
1.0  
0.5  
0.2  
0.0

C10

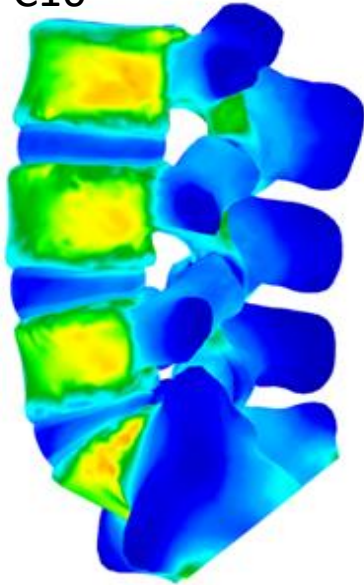10.3  
8.0  
6.0  
5.5  
5.0  
4.5  
4.0  
3.5  
3.0  
2.5  
2.0  
1.5  
1.0  
0.5  
0.2  
0.0

C11

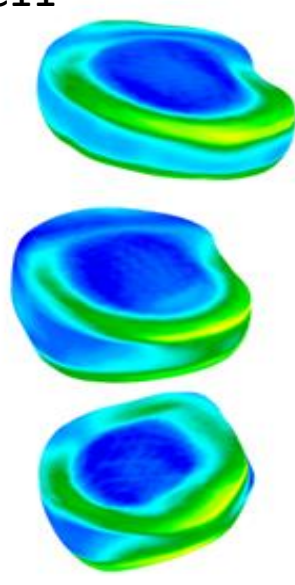5.7  
3.0  
2.5  
2.0  
1.7  
1.5  
1.2  
1.0  
0.8  
0.6  
0.5  
0.4  
0.3  
0.2  
0.1  
0.0

C12

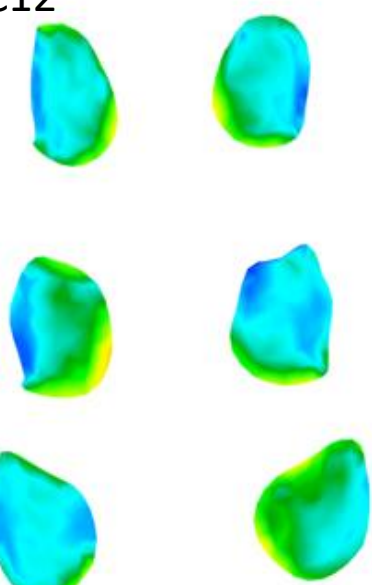2.5  
2.0  
1.8  
1.6  
1.4  
1.2  
1.0  
0.8  
0.6  
0.5  
0.4  
0.3  
0.2  
0.1  
0.0

C13

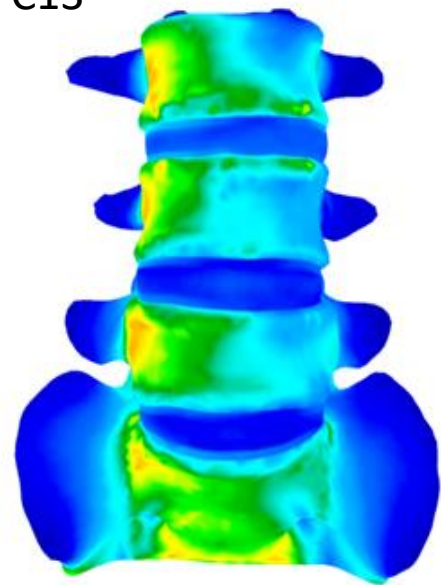10.3  
8.0  
6.0  
5.5  
5.0  
4.5  
4.0  
3.5  
3.0  
2.5  
2.0  
1.5  
1.0  
0.5  
0.2  
0.0

C14

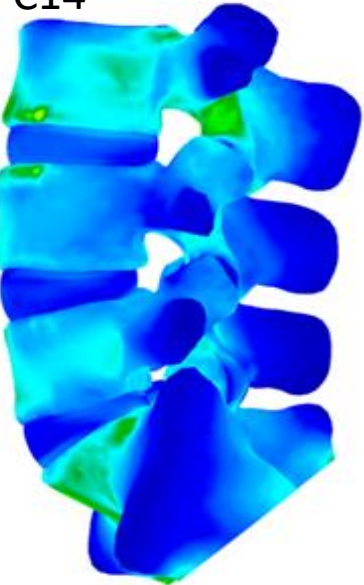10.3  
8.0  
6.0  
5.5  
5.0  
4.5  
4.0  
3.5  
3.0  
2.5  
2.0  
1.5  
1.0  
0.5  
0.2  
0.0

C15

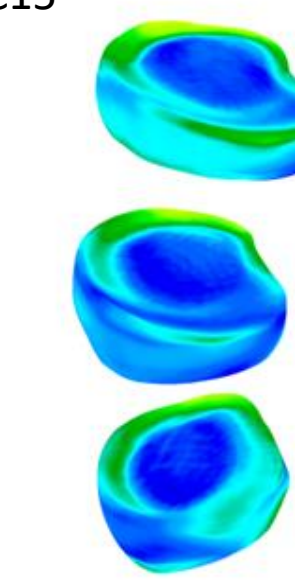5.7  
3.0  
2.5  
2.0  
1.7  
1.5  
1.2  
1.0  
0.8  
0.6  
0.5  
0.4  
0.3  
0.2  
0.1  
0.0

C16

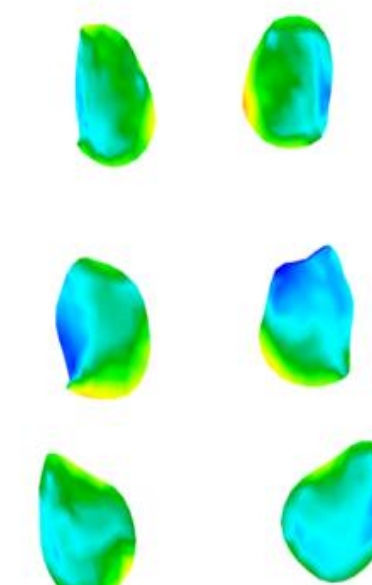2.5  
2.0  
1.8  
1.6  
1.4  
1.2  
1.0  
0.8  
0.6  
0.5  
0.4  
0.3  
0.2  
0.1  
0.0

C17

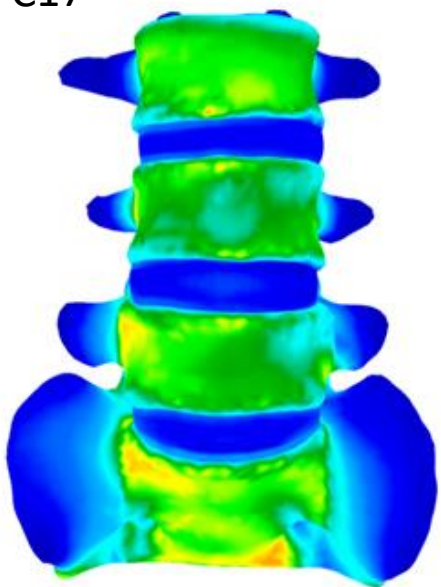

C18

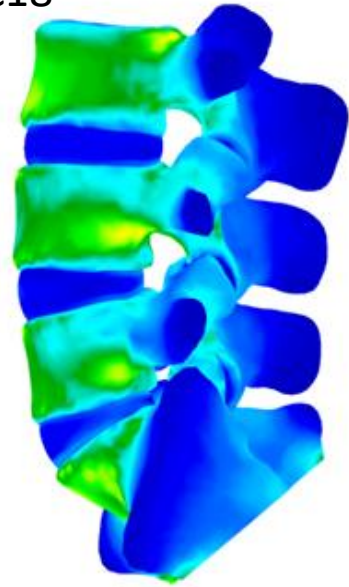

C19

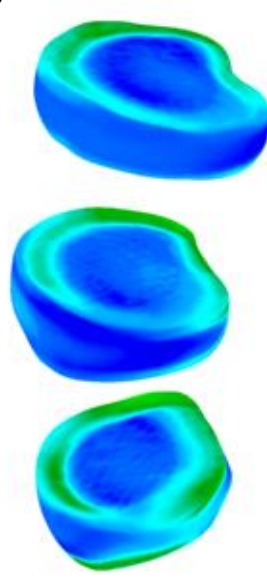

C20

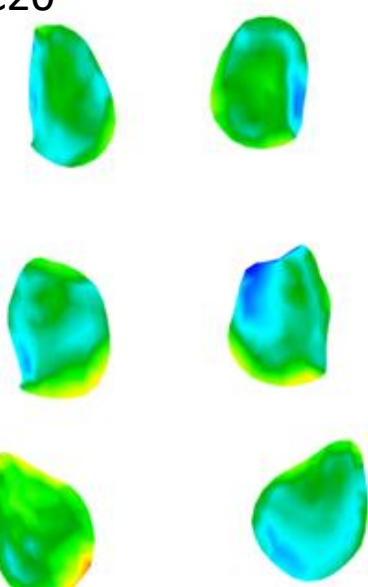

C21

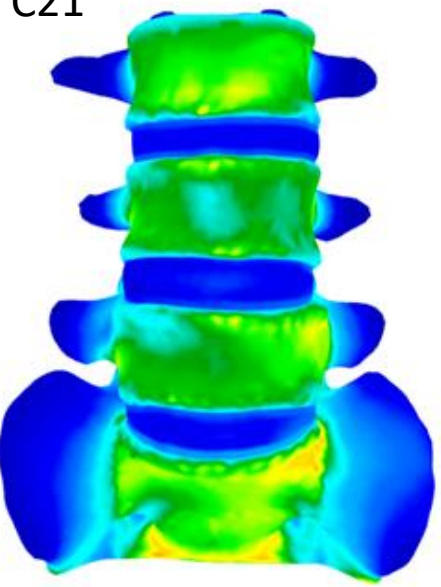

C22

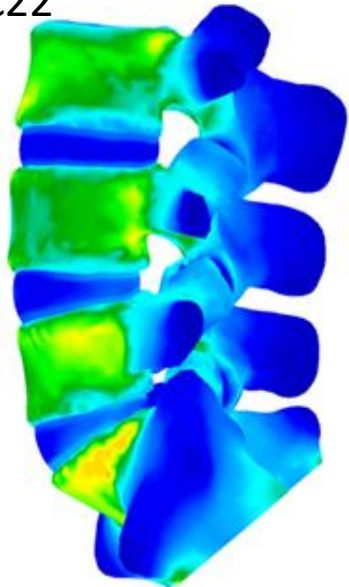

C23

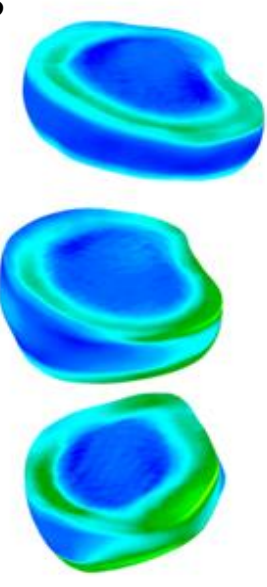

C24

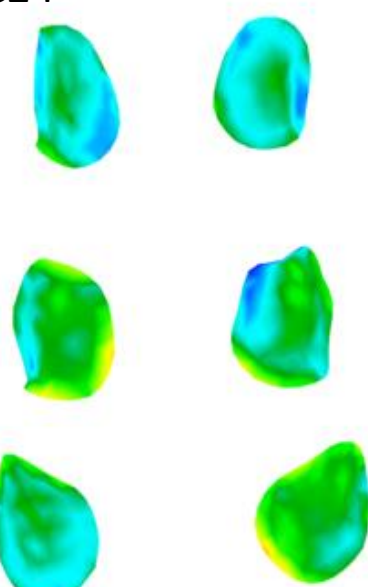

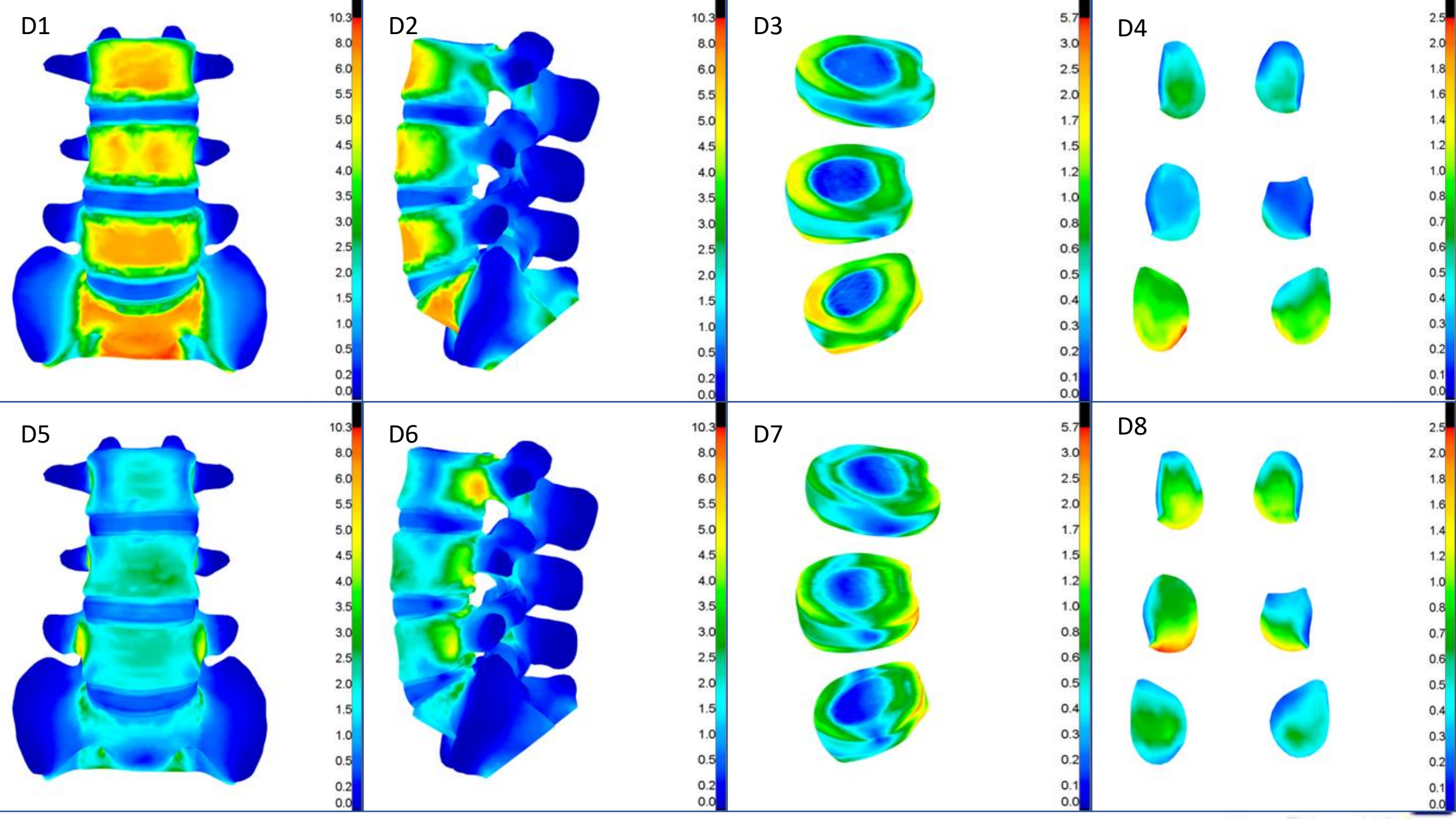

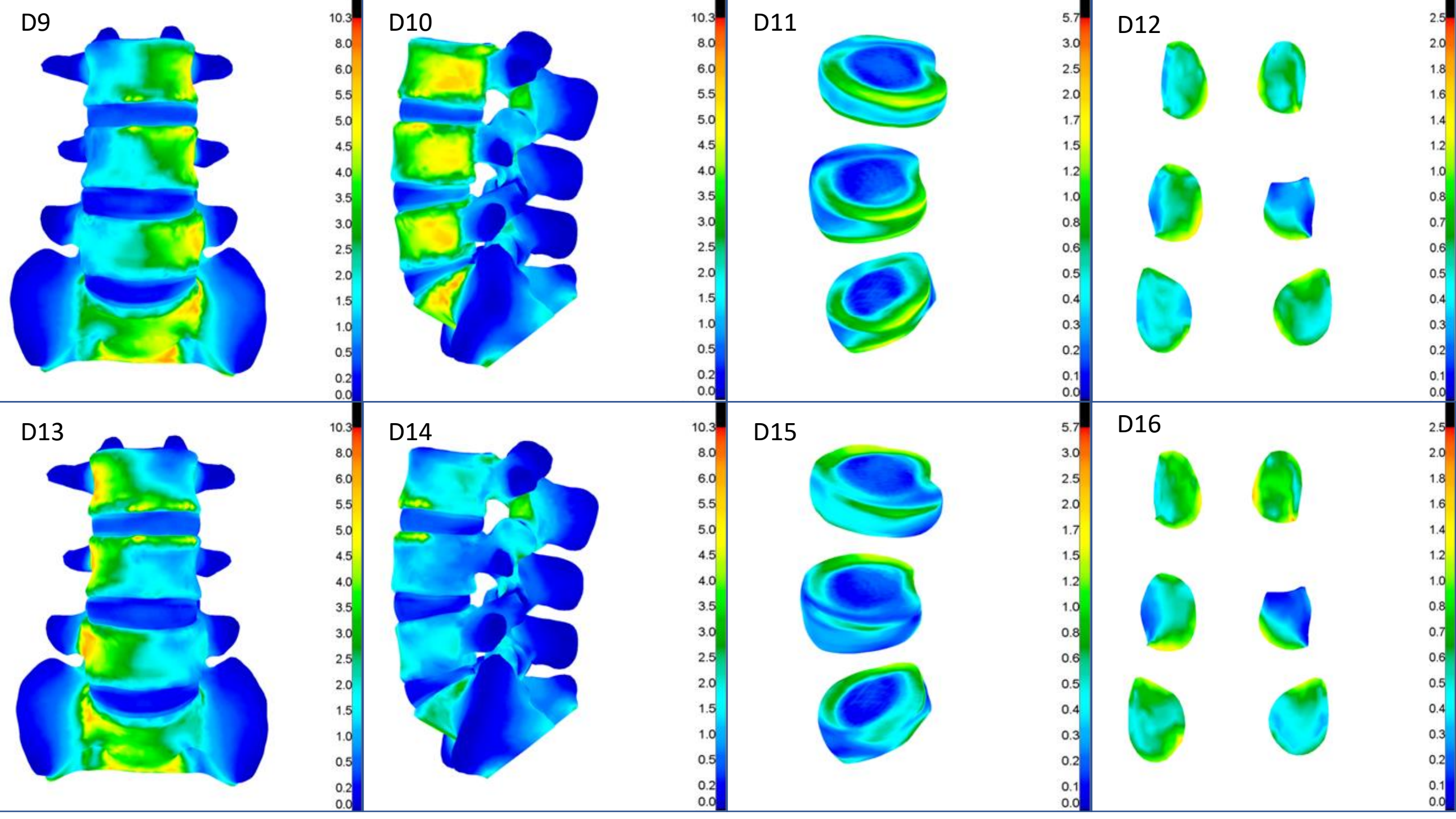

D17

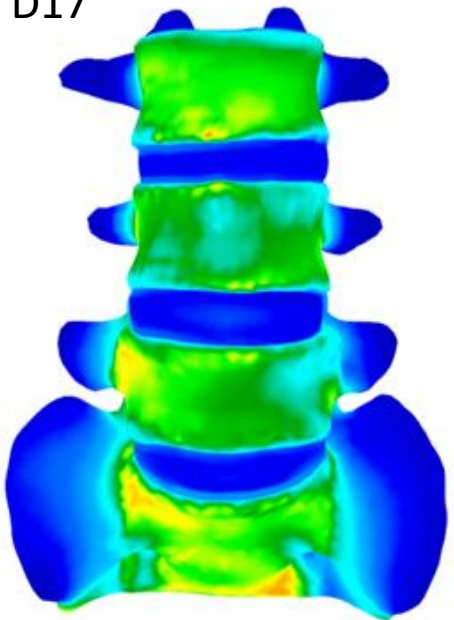10.3  
8.0  
6.0  
5.5  
5.0  
4.5  
4.0  
3.5  
3.0  
2.5  
2.0  
1.5  
1.0  
0.5  
0.2  
0.0

D18

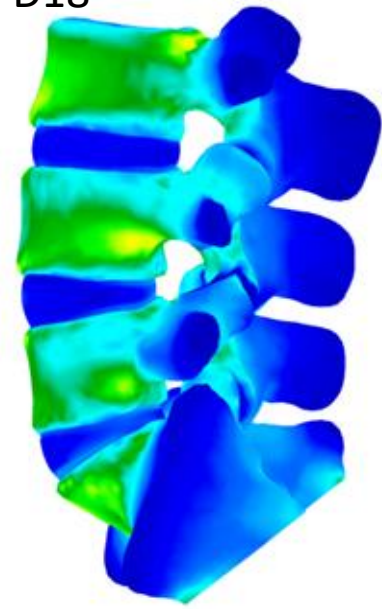10.3  
8.0  
6.0  
5.5  
5.0  
4.5  
4.0  
3.5  
3.0  
2.5  
2.0  
1.5  
1.0  
0.5  
0.2  
0.0

D19

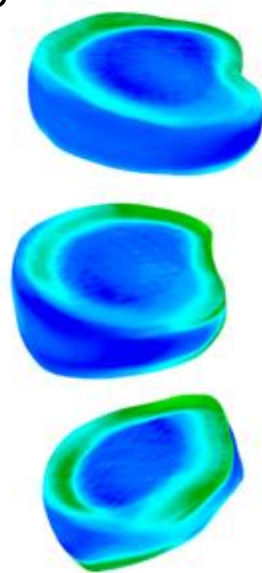5.7  
3.0  
2.5  
2.0  
1.7  
1.5  
1.2  
1.0  
0.8  
0.6  
0.5  
0.4  
0.3  
0.2  
0.1  
0.0

D20

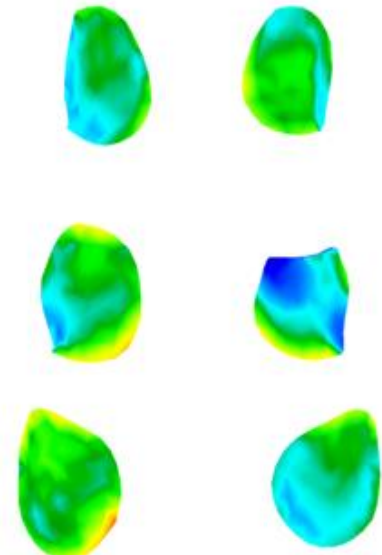2.5  
2.0  
1.8  
1.6  
1.4  
1.2  
1.0  
0.8  
0.7  
0.6  
0.5  
0.4  
0.3  
0.2  
0.1  
0.0

D21

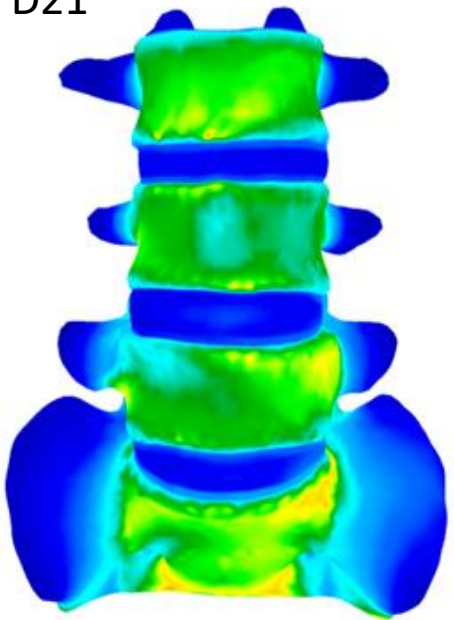10.3  
8.0  
6.0  
5.5  
5.0  
4.5  
4.0  
3.5  
3.0  
2.5  
2.0  
1.5  
1.0  
0.5  
0.2  
0.0

D22

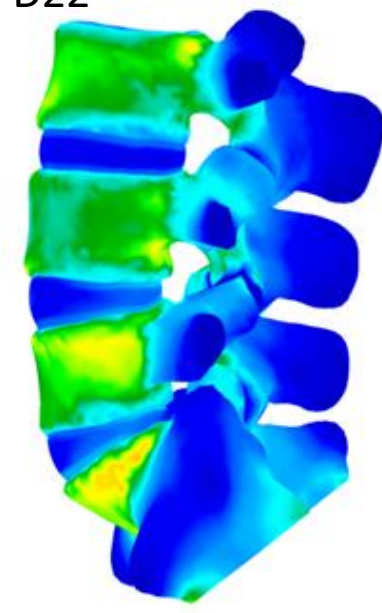10.3  
8.0  
6.0  
5.5  
5.0  
4.5  
4.0  
3.5  
3.0  
2.5  
2.0  
1.5  
1.0  
0.5  
0.2  
0.0

D23

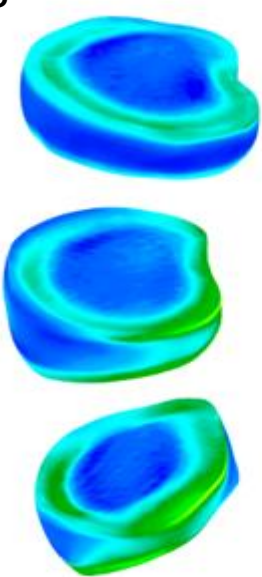5.7  
3.0  
2.5  
2.0  
1.7  
1.5  
1.2  
1.0  
0.8  
0.6  
0.5  
0.4  
0.3  
0.2  
0.1  
0.0

D24

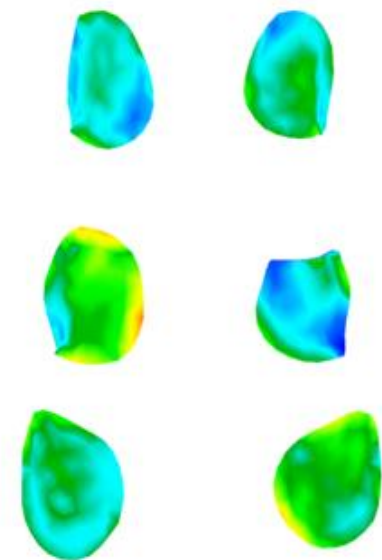2.5  
2.0  
1.8  
1.6  
1.4  
1.2  
1.0  
0.8  
0.7  
0.6  
0.5  
0.4  
0.3  
0.2  
0.1  
0.0

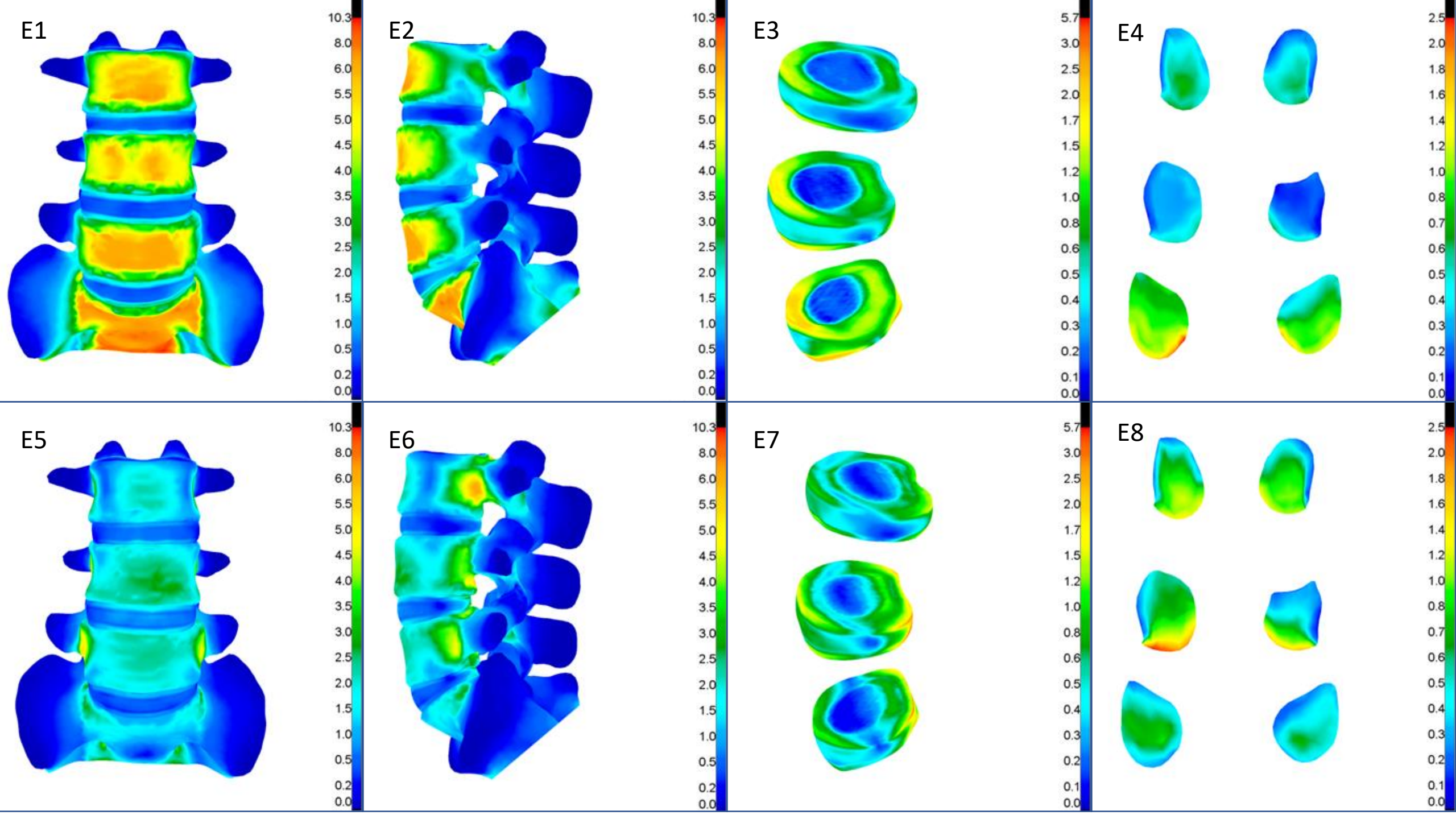

E9

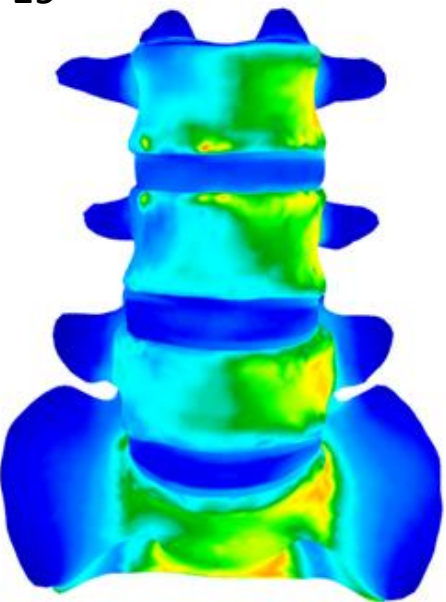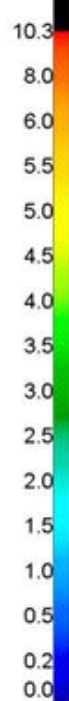

E10

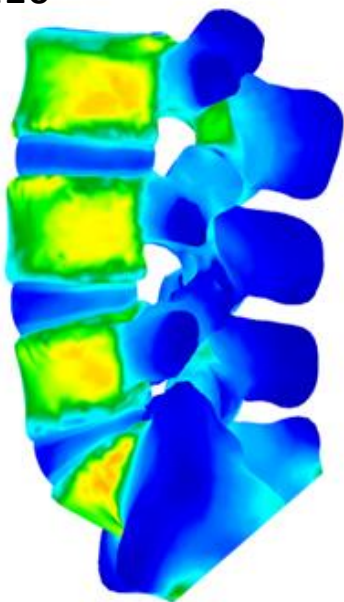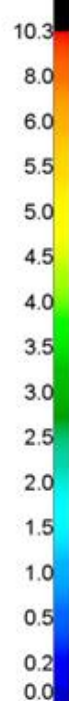

E11

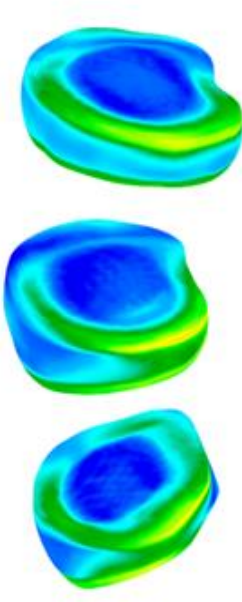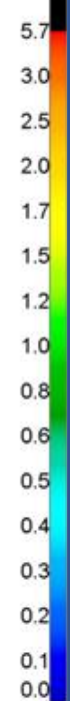

E12

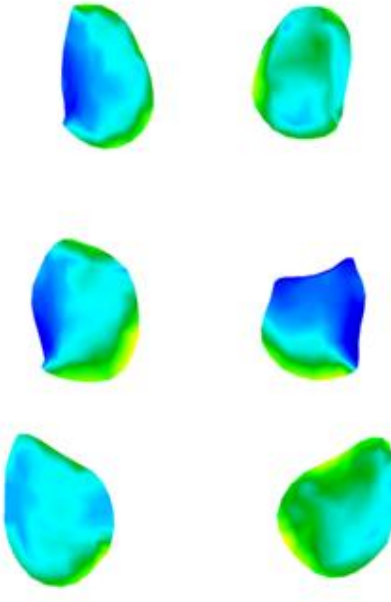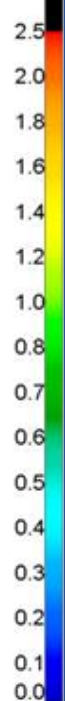

E13

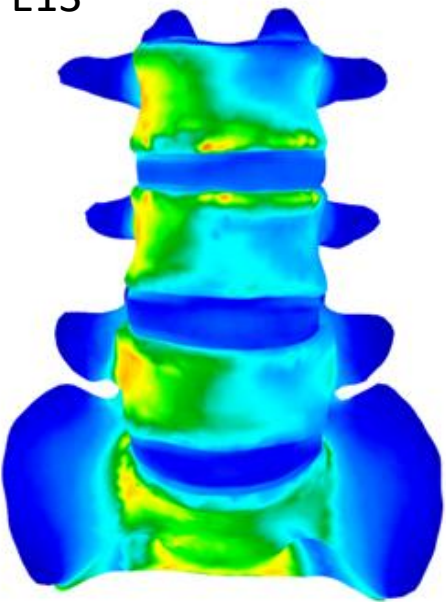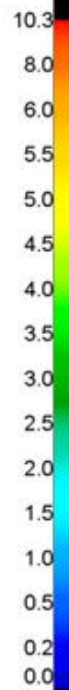

E14

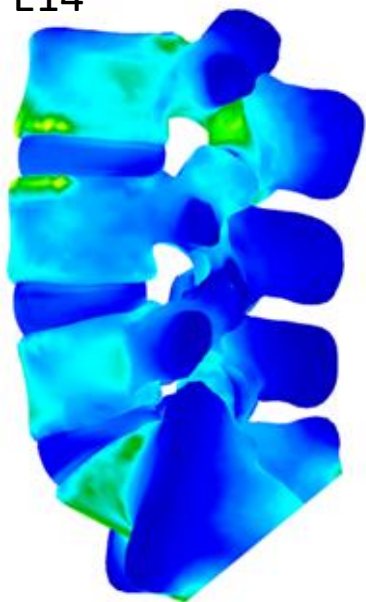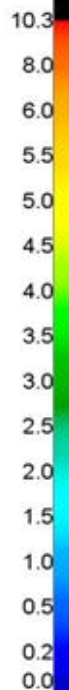

E15

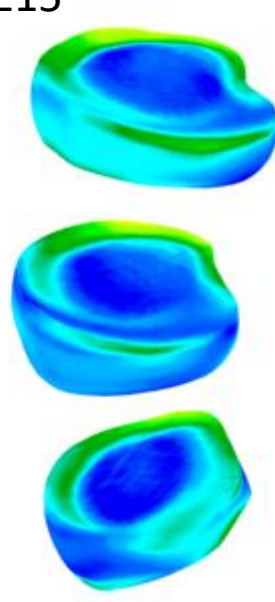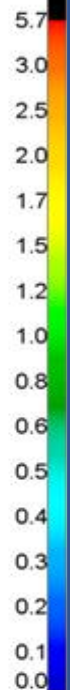

E16

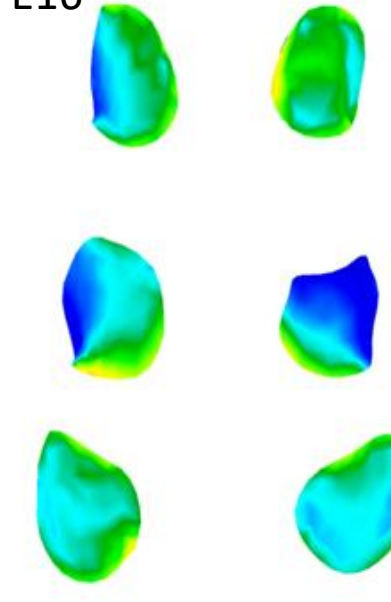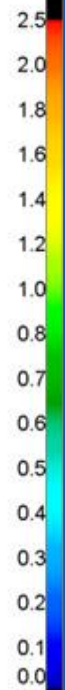

E17

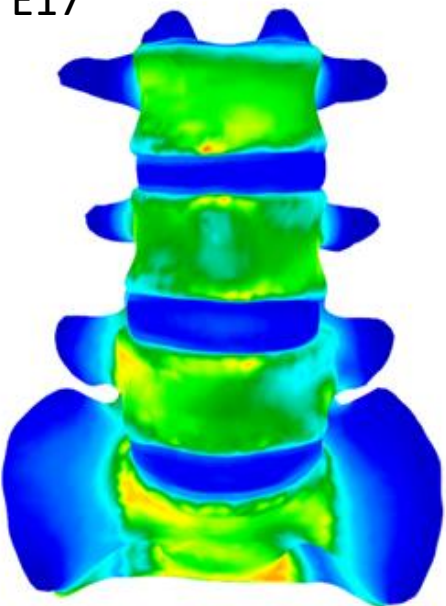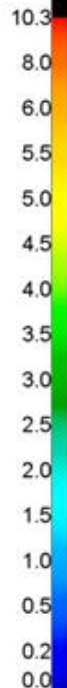

E18

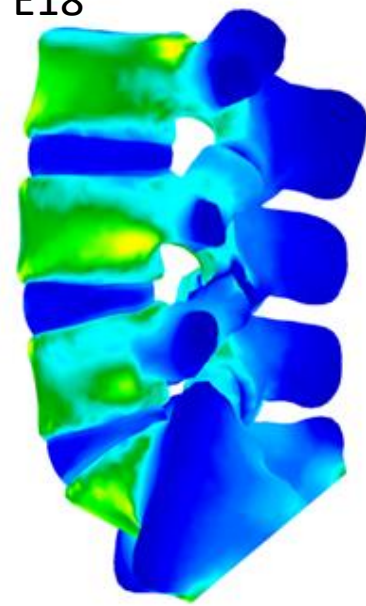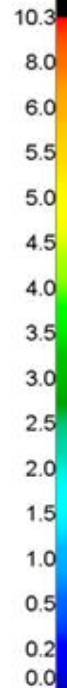

E19

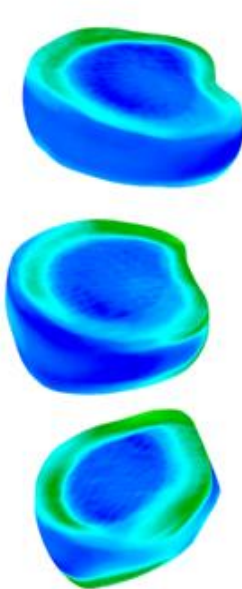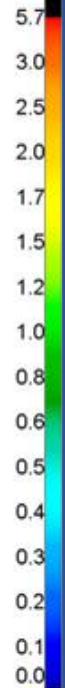

E20

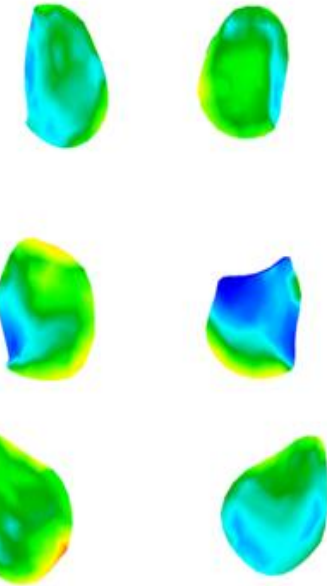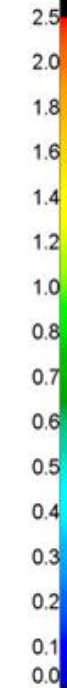

E21

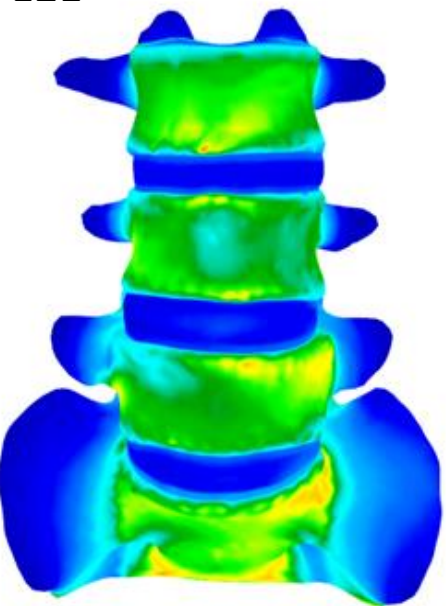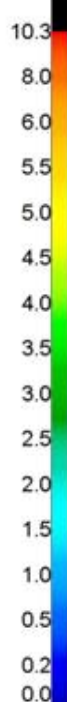

E22

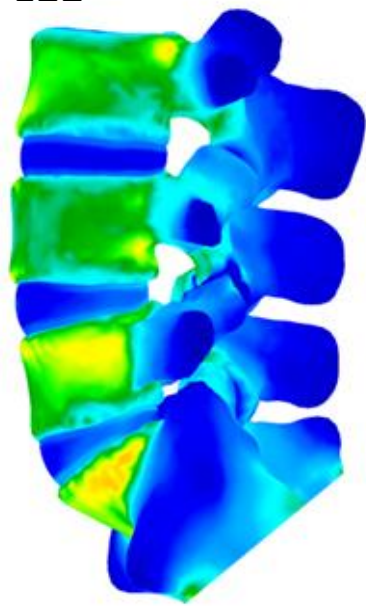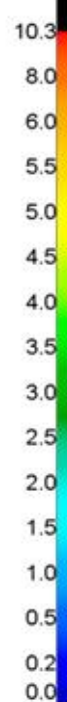

E23

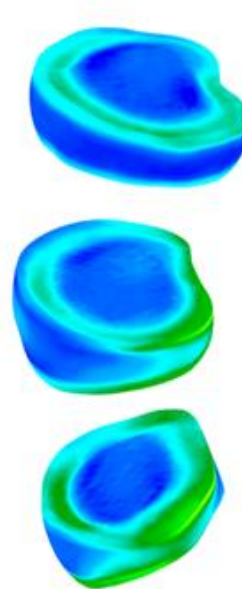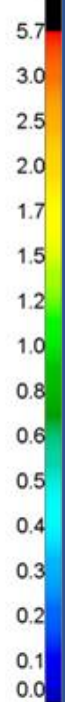

E24

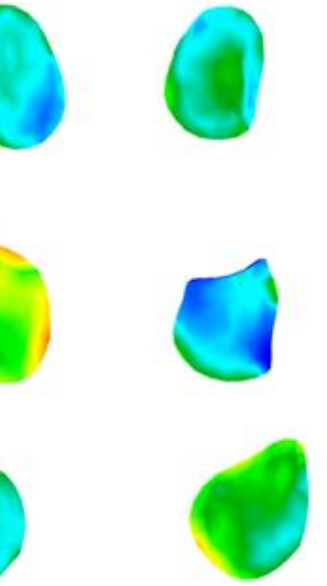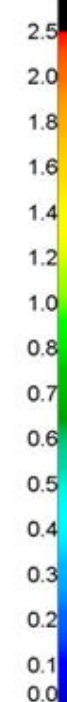

Supplement: Supplementary file 2 — Additional file 2: Figure 1. The von Mises stress nephograms of the vertebral body, intervertebral disc, and facet joints in five groups under six motion. A1-4: Group A under flexion motion; A5-8: Group A under extension motion; A9-12: Group A under left bending motion; A13-16: Group A under right bending motion; A17-20: Group A under left rotation motion; A21-24: Group A under right rotation motion. B1-4: Group B under flexion motion. B5-8: Group B under extension motion; B9-12: Group B under left bending motion; B13-16: Group B under right bending motion; B17-20: Group B under left rotation motion; B21-24: Group B under right rotation motion. C1-4: Group C under flexion motion; C5-8: Group C under extension motion; C9-12: Group C under left bending motion; C13-16: Group C under right bending motion; C17-20: Group C under left rotation motion; C21-24: Group C under right rotation motion. D1-4: Group D under flexion motion; D5-8: Group D under extension motion; D9-12: Group D under left bending motion; D13-16: Group D under right bending motion; D17-20: Group D under left rotation motion; D21-24: Group D under right rotation motion. E1-4: Group E under flexion motion; E5-8: Group E under extension motion; E9-12: Group E under left bending motion; E13-16: Group E under right bending motion; E17-20: Group E under left rotation motion; E21-24: Group E under right rotation motion. [file 13018_2023_3916_MOESM2_ESM.pdf]
